# Passively Ultra Cooling Patch Enabling High‐Efficiency Power‐Water Cogeneration

**DOI:** 10.1002/adma.202505002

**Published:** 2025-08-19

**Authors:** Zhengyi Mao, Yao Yao, Yunhu He, Zhen Yu, Yicheng Han, Junda Shen, Xinxue Tang, Fucong Lyu, Mulin Miao, Yaxin Xu, Zhe Song, Xiaoguang Duan, Yuehong Su, Hongxing Yang, Qiliang Wang, Jian Lu

**Affiliations:** ^1^ Department of Mechanical Engineering City University of Hong Kong 83 Tat Chee Avenue, Kowloon Hong Kong China; ^2^ Renewable Energy Research Group (RERG) Department of Building Environment and Energy Engineering The Hong Kong Polytechnic University Hong Kong China; ^3^ Department of Materials Science and Engineering City University of Hong Kong 83 Tat Chee Avenue, Kowloon Hong Kong China; ^4^ City University of Hong Kong Matter Science Research Institute (Futian) Shenzhen 518045 China; ^5^ School of Chemical Engineering and Advanced Materials University of Adelaide Adelaide 5005 Australia; ^6^ Department of Architecture and Built Environment University of Nottingham University Park Nottingham NG7 2RD UK; ^7^ Centre for Advanced Structural Materials Greater Bay Joint Division Shenyang National Laboratory for Materials Science City University of Hong Kong Shenzhen Research Institute Shenzhen 518057 China; ^8^ Hong Kong Branch of National Precious Metals Material Engineering Research Centre City University of Hong Kong 83 Tat Chee Avenue, Kowloon Hong Kong China

**Keywords:** evaporation cooling, heat and mass transfer, patch, photovoltaics, water‐energy nexus

## Abstract

Photovoltaic (PV) systems, which accounted for 75% of global renewable energy capacity in 2023, are limited by the substantial waste heat generated through the photothermal effect, reducing both electricity generation efficiency and panel longevity. In this work, a passive cooling strategy using an ultra‐cooling patch (UCP) that effectively cools down the PV panel is presented, thus achieving an exceptionally high cooling power of nearly 700 W m^−2^ and facilitating the recovery of over 70% of waste heat for freshwater production. The flexibility and adhesive properties of UCP allow for easy integration with various PV configurations, including flexible panels. In addition, the UCP can be easily reconfigured into a fined structure, enabling modifications to the heat transfer pathway. This passively intensifies the heat dissipation of the PV panel, further enhancing the cooling performance. As a result, a remarkable temperature reduction of 29 °C for the PV panel is achieved, which in turn results in an impressive increase in maximum power density of over 28%. Moreover, outdoor experiments on a ≈1 m^2^ PV panel validated the cooling performance and practical applicability. This study offers a solution with commercial potential to relieve the energy‐water crisis, owing to its high efficiency, scalability, and cost‐effectiveness.

## Graphical abstract

Photovoltaics are the most viable solar energy technology. This work introduces an ultra‐cooling patch (UCP) for passive thermal management of PV, achieving a 28% increase in power density and a water production rate of 2.2 L day^−1^. The enhancement results from the aligned channels and configurable heat transfer pathways of the UCP, which significantly improve thermal dissipation performance.

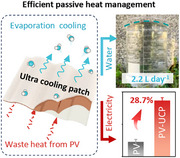

## 1 Introduction

Fresh water and electricity are two of the most essential factors for the further development of modern society.^[^ ^1^, ^2^ ^]^ The growing population, changing climate, and increasing pollution have exacerbated global water and electricity scarcity. The utilization of solar energy for electricity and water generation is widely considered a sustainable solution to water scarcity and electricity shortages. Among all of the solar utilization technologies, photovoltaic (PV) technology is the most viable and widely adopted method for effectively converting solar energy into usable electricity. In the year 2023, the global solar PV cumulative capacity reached a staggering 1500 GW, and which is estimated to contribute ≈22.2% of the total global electricity production.^[^ ^3^, ^4^ ^]^ However, PV cells typically utilize only a specific portion of the solar spectrum for electricity generation (e.g., 300–1100 nm for single‐junction Si cells), resulting in over 70% of the incident energy being converted into waste heat rather than electrical power.^[^ ^5^ ^]^ This accumulated waste heat increases the temperature of the PV panel, thus significantly compromising its power generation performance.^[^ ^6^, ^7^, ^8^ ^]^ Additionally, prolonged exposure to high temperatures also accelerates the degradation of PV panel lifespan.^[^ ^9^, ^10^ ^]^

Recently, there has been a remarkable focus on exploring advanced passive thermal management technologies for PV cooling driven by their appealing advantages, including zero global warming potential, engineering simplicity, and low maintenance costs.^[^ ^11^, ^12^, ^13^, ^14^ ^]^ Passive radiative sky cooling, which dissipates heat through the atmosphere window waveband “8–13 µm” into cold outer space through spectrally selective emitters, has been proven to have a typical cooling power of restricted 40–140 W m^−2^ under clear sky conditions.^[^ ^15^, ^16^ ^]^ By sub‐band gap reflection, the crystalline silicon cools down by 3.8 °C.^[^ ^17^ ^]^ Another attractive method involves integrating water evaporation cooling with a PV panel, offering the dual benefits of enhancing solar utilization efficiency and producing freshwater. This technique typically involves attaching an evaporative layer to the bottom of a PV panel. By utilizing the waste heat from the PV panel for freshwater production and harnessing the generated latent heat to cool the PV panel, there is the potential to improve the power generation performance and prolong the lifespan of the PV panel. In recent years, many evaporation‐cooling layers have been developed, such as hygroscopic hydrogel films,^[^ ^18^ ^]^ self‐pumped multistage membrane structures,^[^ ^19^, ^20^ ^]^ and leaf‐mimetic structures.^[^ ^21^ ^]^ Wang's al. reported on the cooling components of an atmospheric water harvester. Through the atmospheric water sorption and evaporation cycle, an impressive passive cooling power of 295 W m^−2^ was achieved, reducing the temperature of the PV panel by 10 °C under 1.0 kW m^−2^ solar irradiation^[^ ^22^ ^]^

Despite recent advantages, passive thermal management methods still demonstrate lower cooling power than active systems. In addition, the complexity of installation, disassembly, and component composition often hinders easy deployment and large‐scale production. Hence, there remains significant potential to develop an engineering‐simple and user‐friendly passive photovoltaic cooling technology with enhanced cooling capabilities that could significantly boost the power generation efficiency of existing 1500 GW PV installations.

**Figure 1:**
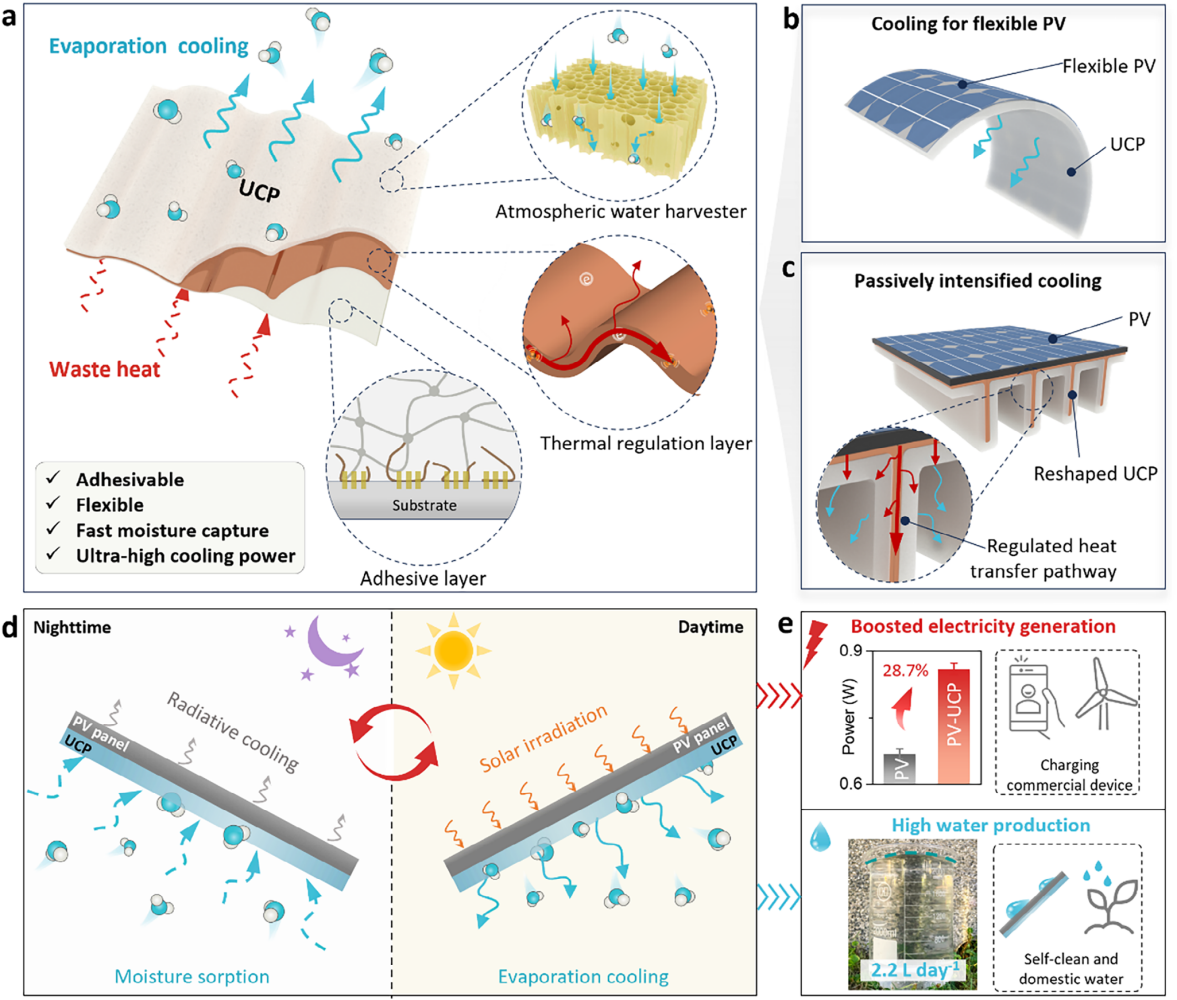
Schematic illustration of the UCP and the PV‐based water‐electricity cogeneration system. a) Structure of the UCP: an atmospheric water harvester for fast moisture capture, a thermal regulation layer to intensify the heat transfer, and an adhesive layer for easy installation and reshaping. b) Schematic of the UCP for cooling flexible PV. c) Reshaped UCP to intensify the passive heat transfer from the PV panel. d) Schematic of moisture capture from air during nighttime, and power‐water cogeneration by solar irradiation during daytime. e) The boosted power generation and water collection performance, with potential applications.

Herein, we present an adhesive and flexible ultra‐cooling patch (UCP) for the highly efficient passive cooling of PV panels while simultaneously producing fresh water. The UCP is composed of 3 layers: an atmospheric water harvester (AWH), a thermal regulation layer and an adhesive layer (**Figure** **1**a). The AWH was prepared using a hydrogel featuring aligned channels and interconnected pores on its wall. After loading with hygroscopic materials, AWH enables rapid absorption of the moisture in the air through such low tortuosity channels. The thermal regulating layer is responsible for adjusting the fast heat transfer pathways. The bottom adhesive layer allows the UCP to adhere tightly and reversibly to various substrates. The flexibility and adhesive properties significantly simplify the installation of the UCP, enabling its application in flexible PV materials (Figure 1b). Moreover, the UCP can be reshaped into fin‐like geometries to expand the interfacial area and further regulate the heat transfer pathways, thereby enhancing passive heat dissipation (Figure 1c). Consequently, this leads to an ultra‐high cooling power density and an enhanced performance in electricity‐water generation. The UCP also demonstrates excellent practical potential, enabling the effective charging of commercial smartwatches and seamless scalability to larger PV panels. By taking advantage of its high efficiency, scalability, and cost‐effectiveness, this research offers a sustainable and potentially commercial solution to alleviate both energy and water challenges.

## 2 Results

### 2.1 Design and Mechanism of the UCP

The mechanism of the PV‐UCP power‐water cogeneration system is shown in Figure 1d. During the nighttime, the UCP captures moisture from the air, with the aligned channel in the UCP and the radiative cooling effect of the PV panel synergistically enhancing the moisture sorption efficiency.^[^ ^23^, ^24^ ^]^ During the daytime, the PV panel generates electricity and heat under solar irradiation. Waste heat can be utilized to vaporize water in the UCP, whereas the latent heat from water evaporation effectively cools the PV panel. This led to simultaneous water production and an increase in electricity generation. As a result, compared with pristine PV panels, our design achieved over 28% enhancement in the maximum power density and could power commercial devices such as cell phones and electric fans (Figure 1e). In addition, after integrating the water collection cover, the generated vapor was collected. The large‐scale system demonstrated a water collection rate of over 2.2 L per day, providing a valuable water source for domestic use and facilitating the self‐cleaning of PV panels.

### 2.2 Synthesis and Characterization of the UCP

**Figure 2:**
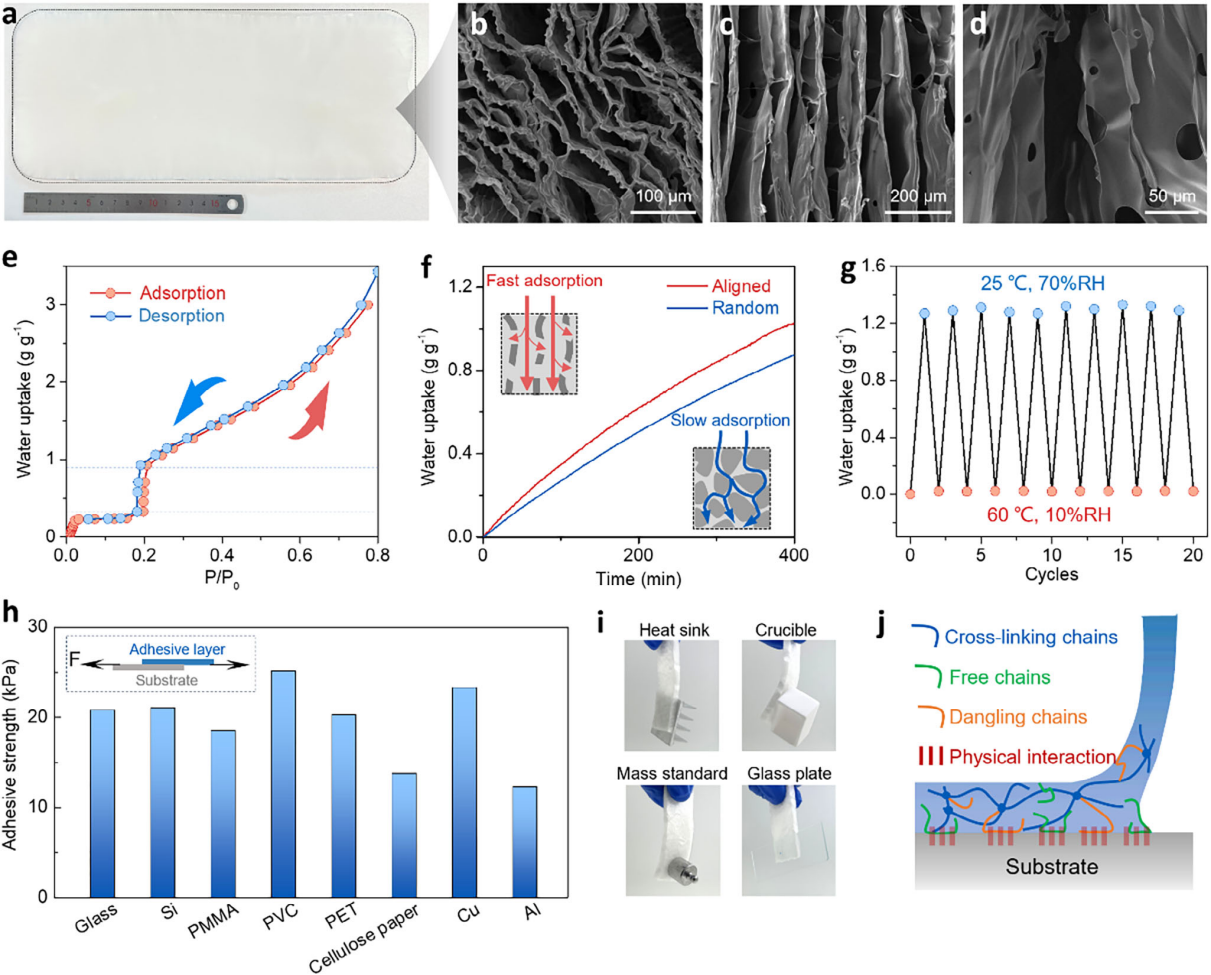
Preparation and characterization of the UCP. a) Optical image of the porous hydrogel sponge (330 × 150 mm). b,c). SEM images of the aligned channels of the hydrogel sponge. d) Micropores on the aligned channels. e) Moisture sorption‐desorption isotherms of the hygroscopic salts under 25 °C at 60% RH. f) The water sorption performance of the AWH with aligned channels and random channels. Insets show the mechanism of the fast sorption of aligned channels. g) Water uptake stability of AWH during repeated adsorption‐desorption cycles under 25 °C at 70% RH for sorption and 60 °C at 10% RH for desorption. h) Lap‐shear adhesion strength of adhesive layer on different substrates. i) Optical images of the patch adhesive on the different materials. j) Schematic illustration of the polymer adhesion mechanism.

The AWH is crucial for the evaporation cooling performance of UCP. To date, various advanced sorbents have been developed such as hygroscopic salts,^[^ ^25^, ^26^ ^]^ Metal‐organic frameworks (MOFs),^[^ ^27^, ^28^, ^29^ ^]^ and polymeric gels.^[^ ^30^, ^31^ ^]^ In this study, hygroscopic salts were selected as sorbents for moisture harvesting because of their exceptional water uptake properties, high energy consumption during desorption, and relatively low cost, making them a particularly advantageous class of materials for this application.^[^ ^13^, ^32^, ^33^ ^]^ Sodium alginate (SA) hydrogel was utilized as the host matrix for the salt.^[^ ^25^, ^34^ ^]^ The porous structure of the hydrogel not only allows for the storage of captured water but it also prevents the aggregation of salt particles during the sorption/desorption cycles. **Figure** **2**a shows the prepared SA hydrogel with dimensions 330 mm × 150 mm. The vertically aligned porous structure of the SA hydrogel skeleton was obtained using directional freeze‐casting (Figure S1, Supporting Information). The prepared SA ink was stirred for over 24 h and rapidly frozen at ‐80 °C on a copper substrate to facilitate directional ice crystallization. Upon the removal of the ice crystals, aligned channels and hierarchical pores were formed (Figure 2b–d). The vertical channel had dimensions ranging from ≈65 to 103 µm, with an average width of ≈89 µm. The corresponding porosity was measured at 96.3%. To prepare the UCP, silicone gel was first painted onto the surface of the porous SA hydrogel skeleton, followed by attachment of the thermal regulation layer. The 150 µm copper sheet was utilized as the thermal regulation layer because of its high thermal conductivity (≈350 W (m K)^−1^), which enables efficient heat transfer from the PV panel to the environment. Next, another layer of silicone gel was added on top of the thermal regulation layer and then placed on plastic wrap at 60 °C for 6 h for cross‐linking. Then, the SA hydrogel skeleton was loaded with hygroscopic salt and dried for an additional 10 h at 80 °C. When in use, the plastic wrap was simply peeled off, and the UCP was attached to the back of the PV panel (Figure S2, Supporting Information). CaCl_2_ is uniformly distributed on the aligned channels (Figure S3, Supporting Information). The SA hydrogel did not exhibit obvious shrinkage during the hydration‐dehydration process (≈3.4%), thus ensuring proper adhesion to the PV panel, even after full dehydration (Figures S4 and S5, Supporting Information). In addition, the SA hydrogel maintained its porous structure even after complete dehydration. This porous structure resulted in a low mass transfer resistance and a large interface between the air and hygroscopic sites, facilitating rapid moisture capture and release (Figure S6, Supporting Information).

The superior water uptake performance of CaCl_2_ (>3 g g^−1^) enables it to capture sufficient moisture from the air (Figure 2e). The water sorption performance of AWH with different pore structures is shown in Figure 2f. The AWH with an aligned channel pore structure exhibited faster sorption kinetics, leading to faster water uptake compared to that with a random pore structure. This enhanced performance is attributed to the low tortuosity of the aligned channel, which reduces the mass‐transfer resistance for water vapor transport. Additionally, the AWH demonstrated rapid vapor capture across a range of humidity conditions (Figure S7, Supporting Information). The water uptake rosed from 0.41 to 1.07 g g^−1^ as the humidity increased from 35% RH to 75% RH over a period of 6 h. After a full night of moisture capture, the AWH retained enough water for evaporative cooling. We then tested the absorption‐desorption cycling stability of the AWH by subjecting it to repeated cycles of absorption at 25 °C and 70% RH, followed by desorption at 60 °C and 10% RH. After 10 cycles, the water‐uptake capacity remained consistent, indicating excellent cycling stability (Figure 2g). By changing the humidity and temperature of the sorption‐desorption cycle, good stability was also observed (Figure S8, Supporting Information).

The adhesiveness of the adhesive layer is essential for assembling and reshaping the UCP. The adhesive layer was prepared by mixing silicone gels of different viscosities and then painting the mixture onto an SA hydrogel, followed by curing at 60 °C for 6 h. The results showed that UCP could adhere strongly to a variety of different materials, including polymers, metals, ceramics, and glass. An adhesion strength of more than 20 kPa was measured on PET, which is commonly used as a photovoltaic backsheet material, demonstrating stable adhesion for PV applications (Figure 2i). Repetitive adhesion tests were also conducted on the actual PV backsheets. Even after 50 cycles of adhesion and detachment, the UCP maintained a high adhesion strength exceeding 20 kPa on the PV backsheet, demonstrating its excellent reversibility (Figure S9, Supporting Information). This stable adhesion was attributed to the physical interaction between the low‐cross‐linked polydimethylsiloxane (PDMS) and the substrate.^[^ ^35^ ^]^ The highly mobile chains, including dangling and free chains, within the adhesive layer allowed easy and conformal contact with the substrate, which increased the van der Waals force and resulted in tight and reversible adhesion of the UCP (Figure 2j). For adhesion between the adhesive layer and AWH, the silicone gel first penetrated the surface of the AWH. Upon curing, interpenetration occurred between the SA and silicone chains at the interface. This resulted in a strong adhesion, which firmly bonded the two layers (Figure S10, Supporting Information). The SEM images of the interface further revealed that the cured silicone gel encased the surface of the cooling layer and was interlocked with its aligned structure, demonstrating its intimate and robust integration.

### 2.3 Cooling Performance of UCP

**Figure 3:**
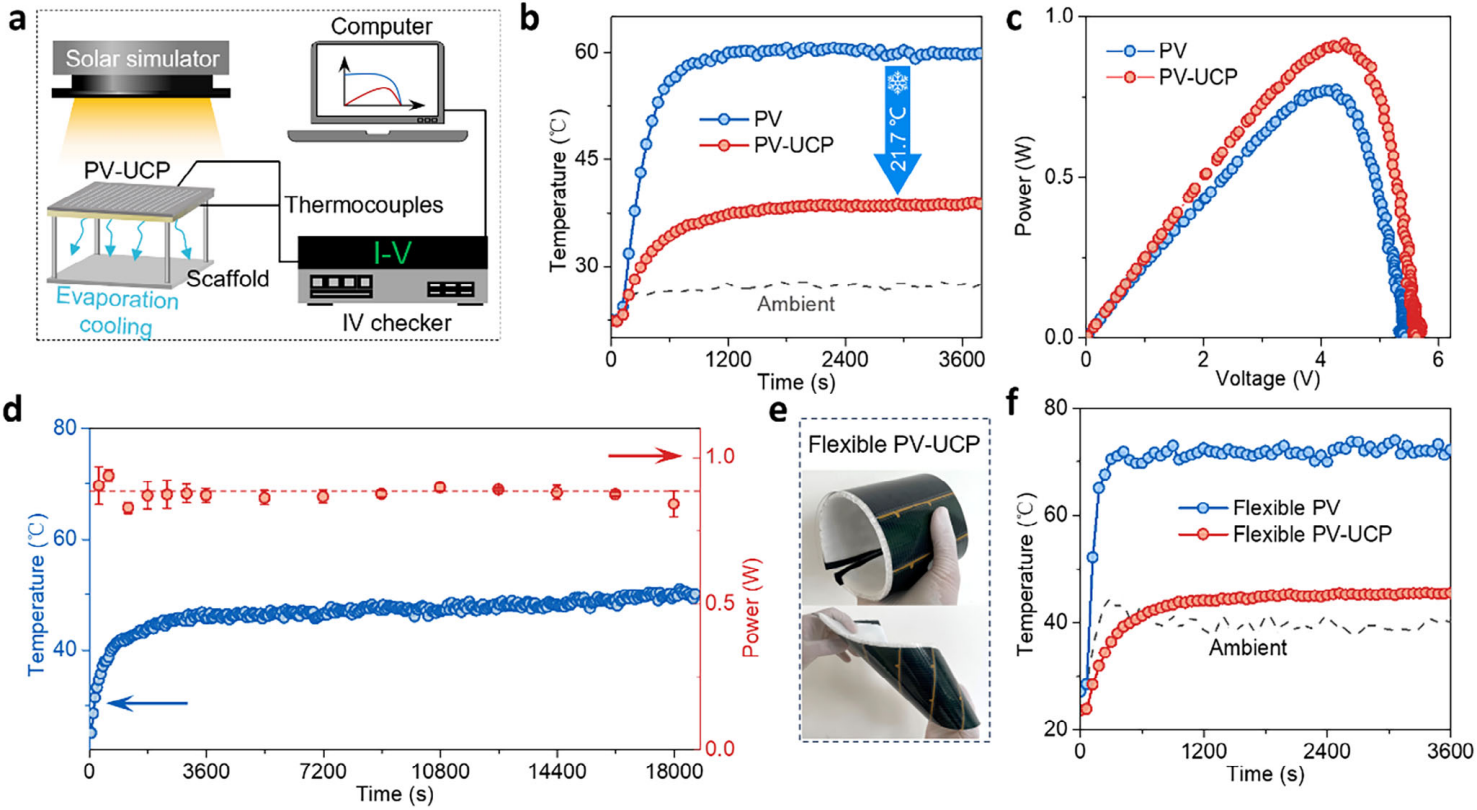
Passive cooling performance of the UCP and power generation performance of PV‐UCP. a) Diagram of the testing platform. The PV‐UCP, with a size of 100 × 100 mm, was placed above a 3D‐printed scaffold to avoid obstructing the evaporation surface. b) Temperature profiles of the pristine PV and PV‐UCP, showing a cooling effect with a temperature reduction of 21.7 °C. c) Power generation performance of the pristine PV and PV‐UCP. d) Long‐term stability assessment of PV‐UCP with a thickness of 10 mm. e) Flexibility demonstration of the PV‐UCP through bending and twisting, with the flexible PV having dimensions of 120 × 290 mm. f) Cooling effect of the UCP on the flexible PV.

In order to measure the cooling effect and power generation performance of the UCP, we designed the experimental setup shown in **Figure** **3**a. The PV panel employed in the experiment had dimensions of 100 × 100 mm, with an effective area of 8000.04 mm^2^. The UCP was adhered to the underside of the PV panel to form PV‐UCP, which was then placed on a 3D‐printed scaffold to facilitate vapor escape (Figure S11, Supporting Information). The PV panel showed strong light absorption at 250–2500 nm (Figure S12, Supporting Information). The experimental temperature profiles of both the pristine PV and PV‐UCP are shown in Figure 3b. Under one sun irradiation, the pristine PV panel reached a temperature of 60.6 °C, whereas the PV‐UCP achieved a significantly lower temperature of 38.9 °C, thus indicating a substantial reduction in operating temperature by 21.7 °C. Correspondingly, the maximum output power density of the PV panel increased significantly from 0.77 W 0.92 W (Figure 3c). The thickness of the UCP critically influenced both the evaporation dynamics and cooling stability in PV‐UCP systems (Figures S13 and S14, Supporting Information). As exposure extended to 3 h, the 2 mm system's limited water content rapidly depleted, causing its evaporation rate to plummet from 0.7 to 0.22 kg m^−2^ h^−1^ and triggering a progressive temperature rise. This degradation was found to have contrasted sharply with the stable performance of thicker UCPs. The 5 and 10 mm systems maintained constant temperatures and evaporation rates throughout the extended operation owing to their greater latent cooling capacity and sufficient water retention. Even after 5 h of continuous operation under one solar intensity at an environmental temperature of 34.8 °C, the PV‐UCP was found to have maintained a temperature that was still 20 °C lower than that of the pristine PV (Figure 3d). This temperature reduction persisted despite prolonged exposure, thus demonstrating the efficient and stable cooling performance of the UCP during the first 5 h. Then, while the cooling performance of the UCP may not remain at optimal levels after 5 h, it can still provide meaningful thermal regulation for the PV panels. Certainly, achieving sustained peak cooling performance through passive evaporation, such as for 8 h, would be ideal, facilitating the application of this technology in most regions worldwide. However, this remains a critical challenge in the field of sorption‐based passive cooling. Future research efforts are necessary to address this issue effectively. Additionally, the flexibility of UCP opens up a promising potential for cooling flexible PV systems. The flexible PV panel with the adhered UCP could still be bent and twisted beyond 360° without causing damage (Figure 3e). The UCP was able to effectively cool down the flexible PV panel by ≈28 °C, resulting in a power density increase of over 72% (Figure 3f; Figure S15, Supporting Information).

To evaluate the potential for the regionalized application of UCP, we tested the performance of the system at different temperatures and humidities. Figure S16 (Supporting Information) depicts the average temperature distribution across different global seasons, showing that the average summer temperature is generally below 38 °C. We compared the cooling effect of the UCP at environmental temperatures of ≈27.2, 34.8 and 40.2 °C. As the environmental temperature increased from 27.2 to 40.2 °C, the temperature of the pristine PV panel increased from 60.2 to 69.1 °C and finally reached 74.7 °C. In contrast, the temperature of the PV‐UCP remained much lower temperatures, registering 38.5 °C, 48.7, and 49.5 °C under the same environmental conditions (Figure S17, Supporting Information). The maximum output power densities of the pristine PV cell and PV‐UCP at different temperatures are shown in Figure S18 (Supporting Information). Across all temperature conditions, the UCP enables boosted power‐generation performance owing to its effective cooling effect. Notably, the performance enhancement was more pronounced at higher ambient temperatures: at 27 °C, the power output improvement was 15.5%, whereas at 40 °C, it reached 25.3%, demonstrating UCP's excellent cooling performance and suitability for use even in high‐temperature environments. Humidity is a critical determinant of cooling performance, primarily because of its effects on atmospheric water harvesting and evaporation rates. To evaluate the cooling performance of UCP at different humidity levels, we selected three humidity conditions for testing: 40% RH, 55% RH, and 75% RH. As shown in Figure S19 (Supporting Information), humidity strongly governs UCP water absorption capacity, directly dictating long‐term cooling stability. While humidity theoretically influences evaporation rate (and thus power density), our study observed only a minor reduction (≈0.5%) when humidity decreased from 75% to 40% RH. This limited effect may stem from the tested humidity range; more pronounced effects are likely at extremes (<30% RH).

### 2.4 Passively Intensified Cooling Efficiency

**Figure 4:**
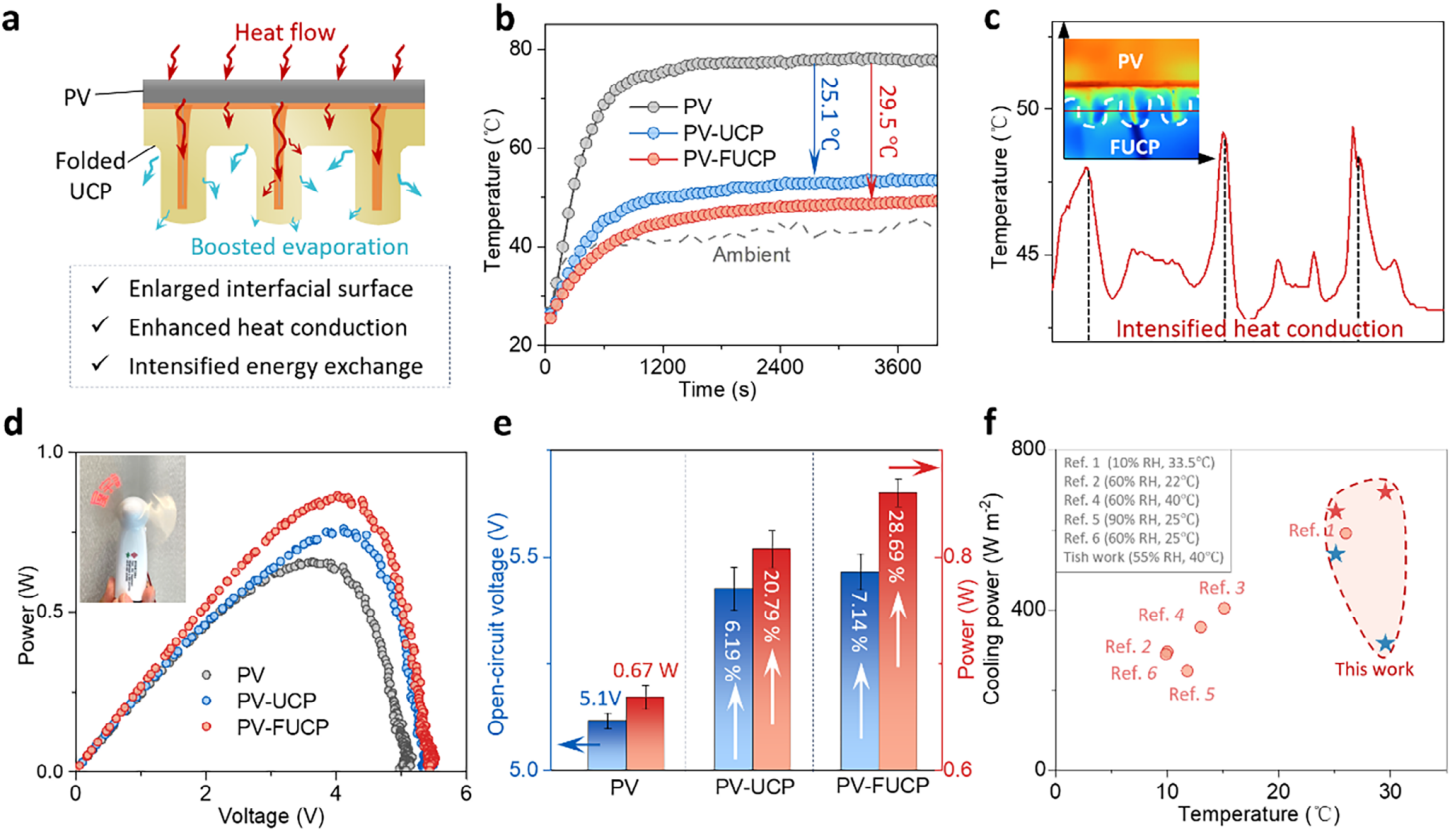
Passively intensified cooling by reshaped UCP. a) Schematics of the folded UCP for enhanced cooling efficiency. b) Comparison of the temperature of the pristine PV panel, PV‐UCP and PV‐FUCP. c) Temperature profile of the FUCP beneath the PV panel, demonstrating the effectiveness of the folded structure in further lowering the temperature. d) Power‐Voltage curves for the pristine PV, PV‐UCP and PV‐FUCP. The inset shows the fan powered by the PV‐FUCP. e) Comparison of the open circuit voltage and power density among the pristine PV, PV‐UCP and PV‐FUCP. The data is presented as mean ± s.d. (n = 3). f) Comparison of the cooling power between the UPC, FUPC and previous works. The red stars correspond to calculations based on the PV projected area, while the blue stars correspond to calculations based on the surface area of the cooling part. Inset shows the testing conditions for cooling power.^[^ ^21^, ^22^, ^36^, ^37^, ^38^, ^39^ ^]^

Cooling performance was further enhanced by reshaping the UCP to take advantage of its flexibility and adhesive properties. Notably, although several sticky cooling patches have been developed recently, they primarily focus on their adhesive properties for attachment to the backs of PV panels, thereby easing installation and application^[^ ^18^, ^37^, ^38^, ^40^, ^41^, ^42^ ^]^ In contrast, the UCP developed in this work incorporates not only the adhesive layer but also a thermal regulation layer that manages rapid heat transfer pathways. **Figure** **4**a illustrates a PV panel with an attached folded UCP (FUCP). By reshaping the UCP, the interface between the thermal regulation layer and AWH, as well as the interface between the AWH and air, are significantly enlarged. This increased surface area enhances the heat flow from the PV panel to the FUCP, intensifying the heat exchange, and thereby boosting the cooling efficiency. As a result, the FUCP reduces the PV panel by 29.5 °C, nearly 5 °C more than the cooling performance of UCP (Figure 4b; Figure S20, Supporting Information). The temperature at the thermal regulation layer was notably higher than that of the AWH at the same height, demonstrating the intensified heat conduction from the PV to the FUCP (Figure 4c). Simultaneously, the evaporated water of the PV‐FUCP reached 10 g, which is 1.4 g more than that of the PV‐UCP. The latent heat from the increased water evaporation effectively consumes the waste heat from the PV panel. Owing to the ultrahigh cooling efficiency of the FUCP, the electrical generation performance of the PV was markedly enhanced, as evidenced by its ability to power a commercial fan (Figure 4d). Specifically, the open‐circuit voltage of the PV panel increased by 6.19% with the UCP and 7.14% with the FUCP (Figure 4e). Additionally, the maximum power density of the PV‐FUCP system showed significant improvements, increasing by 28.69% when compared to the standalone PV panel and almost 8% over the PV‐UCP. We compared the cooling capabilities of the UCP with those of previously reported sticky patches (Table S1, Supporting Information). The developed UCP outperformed most of the reported cooling patches in terms of temperature reduction, power enhancement, and stability, underscoring the significant benefits of the FUCP design in improving cooling efficiency and power generation performance. To tangibly demonstrate the enhanced power generation capability afforded by the FUCP design, we conducted a practical test by charging two smartwatches (Band 8, HUAWEI) using the PV‐FUCP and PV‐UCP under 1 sun, respectively (Figure S21a, Supporting Information). After 10 min of charging, the smartwatch charged by the PV‐FUCP achieved a 19% battery increase, whereas the one charged by the PV‐UCP resulted in only resulted in a 15% increase (Figure S21b and Video S1, Supporting Information). This demonstrates the considerable advancement of the passive ultra‐cooling strategy for practical implementation.

We have summarized and compared the performance of the UCP with that of 20 previously reported related systems (Table S2, Supporting Information). As evaporation cooling performance is strongly influenced by test conditions such as temperature and humidity, we selected representative studies that employ evaporation layers for PV cooling. To enable a more accurate and condition‐aware evaluation, the corresponding testing conditions of humidity and temperature for cooling power are compiled and presented in Figure 4f and Table S3 (Supporting Information).^[^ ^21^, ^22^, ^36^, ^37^, ^38^, ^39^ ^]^ The cooling power density of the UCP and FUCP was calculated using two commonly reported reference areas: the projected area of the PV and the surface area of the cooling structure. According to the projected area calculation, the cooling power densities were 645 W m^−^^2^ for the UCP and 692 W m^−^^2^ for the FUCP. In contrast, using the surface area calculation, the cooling power densities were 538 W m^−^^2^ for the UCP and 315 W m^−^^2^ for the FUCP. Although the FUCP has a lower cooling power density due to its larger surface area, it still offers superior overall cooling performance for the PV panel because of its enhanced heat dissipation capability. This superior cooling performance was mainly attributed to three factors: i) Efficient mass transfer in the aligned channel of the AWH. The low tortuosity of the aligned channel minimizes the diffusion resistance, enabling fast sorption/evaporation kinetics (Figures S22 and S23, Supporting Information); ii) the high thermal conductivity of the thermal regulation layer and the large interface between the thermal regulation layer and the AWH, which allows for rapid transfer of waste heat from the PV panel to the UCP; and iii) a large interface between the AWH and the surrounding air, which facilitates the escape of water vapor and boosts latent heat release, further cooling the PV panel (Figure S24, Supporting Information). To quantitatively analyze the crucial role of FUCP in cooling the PV panel, we calculated the heat flow of three 3 systems: pristine PV, PV‐UCP, and PV‐FUCP (Figure S25 and Note S3, Supporting Information). Compared with the pristine PV panel, over 69% of the waste heat was removed through the rapid water evaporation of the UCP. Moreover, the newly designed FUCP passively contributed an extra 0.95 W of latent heat to further cool the PV panel, which simultaneously boosted the water production and power generation efficiency.

The impact of different structural parameters, including the spacing distance, length of the folded area, thickness of the thermal regulation layer, and thermal conductivity of the thermal regulation layer, on the evaporation performance was studied through simulations. The simulation results show that adjusting the structural parameters, such as increasing the length and density of the folded interfacial area or increasing the thermal conductivity/thickness of the thermal regulation layer, can significantly improve the cooling effect (Figures S26–S29, Supporting Information). However, some of these adjustments significantly increase material costs and manufacturing difficulty. Therefore, practical deployment and application require further evaluation of the trade‐off between cost and performance.

### 2.5 Outdoor and Large‐Scale Deployment

**Figure 5:**
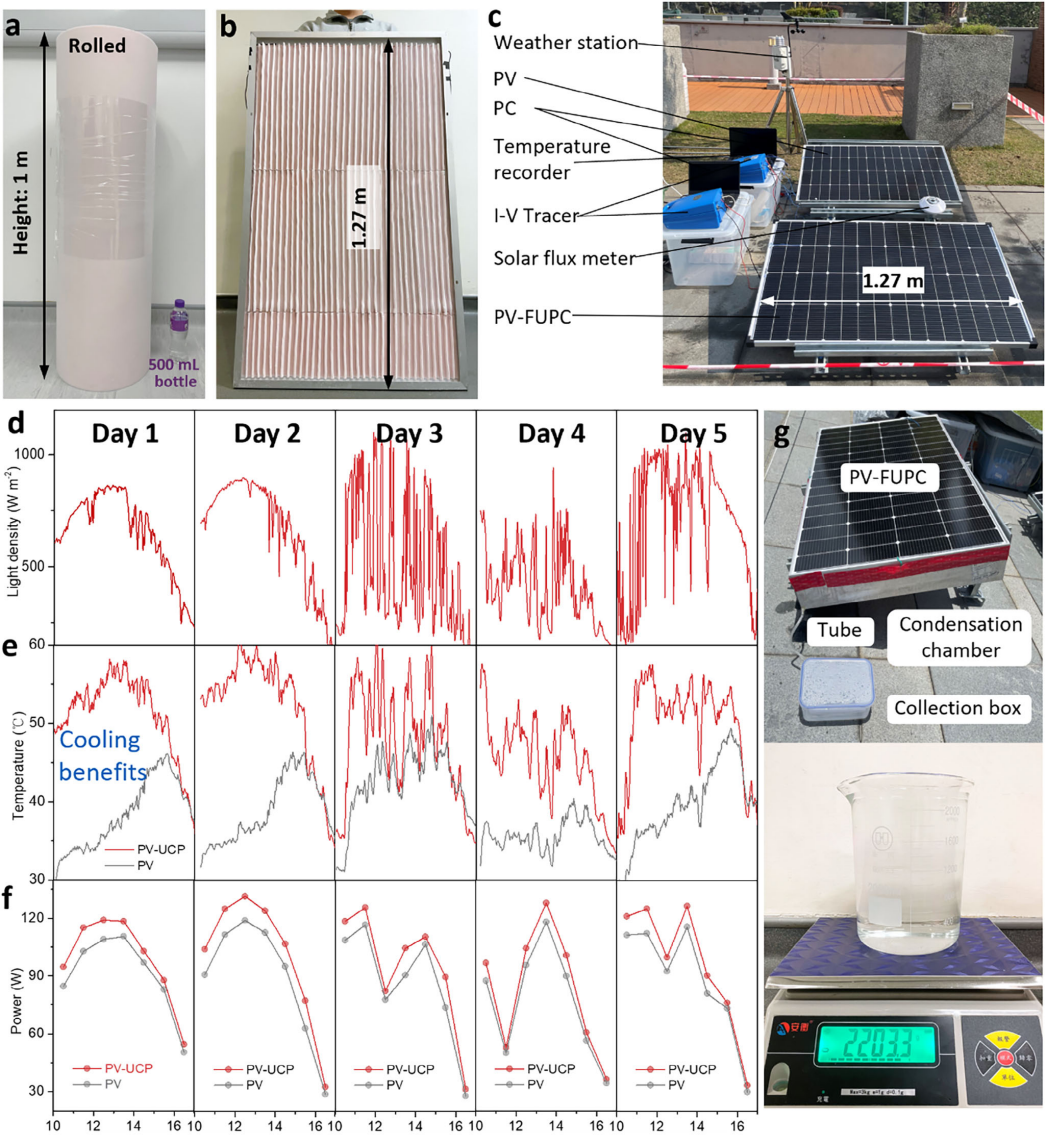
Outdoor and large‐scale deployment. a) Rolled UCP with a size of 2000 mm × 1000 mm. b) Prepared PV‐FUCP unit with dimensions of 1270 mm × 760 mm. c) Outdoor setup of PV‐FUCP and referenced pristine PV test. d) Light intensity during the 5‐day test. Temperature e) and maximum power density f) of the PV and PV‐FUCP under outdoor conditions. g) Water collection device for PV‐FUCP.

We further evaluated the effectiveness of our strategy by utilizing flexible adhesive patches to passively enhance heat dissipation during practical and large‐scale deployment. Commercially available melamine sponge and copper tape were utilized in order to fabricate a large UCP with dimensions of 2000 mm × 1000 mm, which could be easily rolled for convenient storage and transportation (**Figure** **5**a; Figure S30, Supporting Information). The reshaped UCP was adhered to the backplane of a commercial PV panel (1270 mm × 760 mm) to form the PV‐FUCP (Figure 5b; Figure S31, Supporting Information). The outdoor experiments were carried out at The Hong Kong Polytechnic University (22°18′15″N, 114°10′45″E) for 5 days from April 12 to April 16. Figure 5c shows the setup of the outdoor tests for the large‐scale pristine PV panel and the PV‐FUCP. The environmental conditions, including light density, temperature, humidity, and wind speed, were continuously recorded (Figure 5d; Figure S32, Supporting Information). The temperature and power density of the pristine PV panel and the PV‐FUCP were measured to evaluate the effectiveness of the strategy for achieving PV cooling and power output enhancement in practical large‐scale applications. Under natural sunlight, the FUCP significantly reduced the operation temperature of the PV panel by 21.2 °C and 24.7 °C over the first two days (Figure 5e). Owing to the cooling effect, the power‐generation performance increased from 102.9 W 115.1 W (Figure 5f). The results also showed that cloudy weather can reduce the cooling effectiveness, as both the PV temperature and power output were lower under cloudy conditions. The successful implementation of the UCP on a 1 m^2^ PV panel, which is the typical size used in distributed photovoltaic systems, demonstrates the scalability potential of this technology. For broader deployment, such as in distributed PV arrays, the modular nature of PV systems facilitates system‐level scalability. However, the scalability of this technique still faces some challenges, such as installation and maintenance issues after scaling up. Although the adhesive design facilitates installation, factors such as labor costs, maintenance methods, and maintenance cycles in large‐scale applications should be considered in future real‐world testing scenarios. Additionally, potable water can be obtained using this strategy. By integrating a condensation chamber behind the PV panel, more than 2.2 kg of water could be collected during the daytime, which could be used for domestic consumption and self‐cleaning of the PV panels (Figure 5g; Figure S33, Supporting Information). It should be noted that the integrated condensation chamber may reduce the evaporation performance, resulting in decreased cooling efficiency. The tradeoff between the higher water production and greater power density should be considered based on the specific application context, such as whether water scarcity or electricity demand is more critical.

## 3 Discussion

In summary, we have developed a flexible and adherable patch with ultra‐efficient passive cooling capabilities that can significantly boost the power generation of PV panels while simultaneously enabling freshwater production. The PV panel temperature was significantly lowered by 29 °C, and its power density was accordingly improved by 28% owing to the effective heat dissipation provided by the UCP. The flexibility and adhesive properties of UCP offer advantages such as long‐term durability, ease of deployment, and applicability to flexible PV systems under various temperature conditions. Moreover, the scalability and practical potential of our strategy were successfully demonstrated by attaching the UCP to a 1 m^2^ PV panel in a real‐world setting.

Despite the progress made, the actual application and widespread adoption of this technology require further exploration. The current evaluation of the real application of PV‐UCP systems has certain limitations. First, the environmental impacts at real application sites, including high temperatures, drought, wind speeds, and extreme weather conditions, should be considered. For example, extremely low humidity can impede moisture capture, significantly affecting both cooling power and stability. This limitation presents a fundamental challenge in sorption‐based passive cooling technologies. Potential mitigation strategies include employing advanced sorption materials (e.g., MOFs) engineered for efficient atmospheric water harvesting in low‐humidity environments;^[^ ^27^, ^43^ ^]^ however, this may increase material costs and reduce the replenishment capacity at high humidity. Therefore, the selection of technology for PV cooling applications requires a careful assessment of site‐specific climatic conditions. Secondly, cost‐effectiveness should be considered. The calculated costs are shown in Table S4 (Supporting Information). UCP shows objective benefits for rooftop PV in Hong Kong, where the photovoltaic electricity price and humidity levels are very suitable for UCP applications. However, in other locations, particularly where electricity prices are lower, the economic viability of the performance must be considered. Third, there is a tradeoff between water production and enhanced power generation. When a vapor condenser was attached, the water evaporation rate reduced owing to the closed chamber. Consequently, the cooling power density of the UCP decreased from 538 to 439 W m^−2^. In practical applications, it is crucial to strike a balance between water and electricity values when navigating this tradeoff.

## Conflict of Interest

The authors declare no conflict of interest.

## Supporting information



Supporting Information

Supplemental Video 1

## Data Availability

The data that support the findings of this study are available in the supplementary material of this article.
