# Supplementary material for: Passively Ultra Cooling Patch Enabling High‐Efficiency Power‐Water Cogeneration
Source: Adv Mater. 2025 Aug 19;38(2):e05002. doi: 10.1002/adma.202505002 (PMC12783949; doi:10.1002/adma.202505002)
Supplement: Supplementary file 1 — Supporting Information [file ADMA-38-e05002-s002.docx]

**Passively ultra cooling patch enabling high-efficiency power-water cogeneration**

**Table of contents**

**Supplementary Notes 1 to 3**

**Supplementary Figures 1 to 33**

**Supplementary Tables 1 to 4**

**Supplementary References**

**Supplementary Note 1. Materials and Experimental tests**

**S1. Preparation of the UCP**

In order to prepare the atmospheric water harvester (AWH), 3 wt. % sodium alginate (SA, Aladdin) was first dissolved in DI water and stir for 24h. The solution was then poured into a customized copper mold for directional freezing. Finally, the evaporator was lyophilized at −55 °C at a vacuum pressure of 0.01 mbar for 48 h (FD-1C-50+, Biocool). The adhesive layer was formulated by mixing Sylgard 184 (prepolymer:curing agent = 30:1) and SE 1700 (prepolymer:curing agent = 30:1) at a 1:1 mass ratio to achieve stable adhesion and viscosity. the adhesive ink was then applied onto the AWH, followed by placing a 150 μm copper sheet on top. The other side of the copper sheet was coated with adhesive ink. Subsequently, the prepared sample was cured at 60℃ for 6 h.

**S2. Characterization**

Scanning electron microscopy images were recorded using a scanning electron microscope (Quanta 450 FEG), before which a gold layer was deposited on the fractured section to increase the conductivity. The absorption and reflectance spectra were measured using an ultraviolet-visible-light-near-infrared spectrometer (Solidspec-3700). The thermal images were recorded using an infrared thermal imaging camera (Fluke Ti400). Lap shear tests were conducted using a mechanical testing device (CellScale). An adhesion area of 1◊1 cm was prepared. The shear strength was determined by dividing the maximum force by the adhesion area. Moisture sorption was measured at constant temperature and humidity. A numerical simulation of the heat conduction was performed using the finite element method in COMSOL Multiphysics v5.6.

**Supplementary Note 2. Experimental conditions for power–water generation tests**

The moisture sorption and desorption performance was recorded using an electrical balance (Zhuojing, China). Moisture sorption was measured at constant temperature and humidity. Flexible PV were purchased from Kunshan Jingyang PV Technology Co., Ltd. The PV panels were purchased from Dongguan Sunworld Co., Ltd. The experiments were performed under 1 sun illumination (Honle UV Technology). Light intensity was measured using an optical power meter (MS-802, EKO Instruments). The open-circuit voltage and power density were recorded and calculated using an I-V checker (MP-11H, EKO Instruments). Temperature was measured using a thermocouple (Runjiang Instruments). The ambient temperature was regulated by air conditioning. The test system was enclosed in an acrylic box to prevent air movement from affecting the results. Unless otherwise specified, the PV system was measured without a condensation box, and the testing humidity was set to approximately 55% RH. Before testing, the UCP was placed under laboratory conditions overnight in order to capture the vapor. The amount of water stored in the UCP with a thickness of 5 mm was approximately 0.298 g cm^-2^.

**Supplementary Note 3. Energy balance analysis of PV-based systems**

To quantitatively analyze the crucial role of UCP and FUCP, the energy balance in the pristine PV panel, PV-UCP and PV-FUCP was calculated. The PV system was suspended on a 3D-printed scaffold, and conductive heat loss from the support was negligible. Energy consumption includes conventional, radiation, evaporation, and power generation. Energy input was obtained from solar irradiation.

**(1) Energy input**

The input solar power (P_input_) was calculated using the following equation (1):

| $P_{input}=\alpha C_{opt}q_{i}A_{pro}$ | (1) |
| --- | --- |

Where α is the optical absorption coefficient, C_opt_ represents the optical concentration; and qi is the normal direct solar irradiation (1 kW m^−2^ for 1 sun at AM 1.5), A_pro_ is the project area of the absorber.

**(2) Radiation loss**

The radiation loss of the pristine PV ($P_{rad,PV}$), PV-UCP ($P_{rad,PV-UCP}$), and PV-FUCP ($P_{rad,PV-FUCP}$) can be calculated by equation (2), (3) and (4), respectively,

| $P_{rad,PV}= {A_{pro}\varepsilon}_{1}\sigma{(T}_{1}^{4}-T_{0}^{4}$*)+*${A_{sid,PV}\varepsilon}_{2}\sigma{(T}_{2}^{4}-T_{0}^{4}$*)+*${A_{back,PV}\varepsilon}_{3}\sigma{(T}_{3}^{4}-T_{0}^{4}$*)* | (2) |
| --- | --- |
| $P_{rad,PV-UCP}= {A_{pro}\varepsilon}_{1}\sigma{(T}_{4}^{4}-T_{0}^{4}$)+${A_{sid,PV-UCP}\varepsilon}_{4}\sigma{(T}_{5}^{4}-T_{0}^{4}$)+${A_{back,PV-UCP}\varepsilon}_{4}\sigma{(T}_{6}^{4}-T_{0}^{4}$) | (3) |
| $P_{rad,PV-FUCP}= {A_{pro}\varepsilon}_{1}\sigma{(T}_{7}^{4}-T_{0}^{4}$)+${A_{sid,PV-FUCP}\varepsilon}_{4}\sigma{(T}_{8}^{4}-T_{0}^{4}$)+${A_{back,PV-FUCP}\varepsilon}_{4}\sigma{(T}_{9}^{4}-T_{0}^{4}$) | (4) |

Where σ is the Stefan–Boltzmann constant, *A _sid,PV_*, *A _sid,PV-UCP_*, *A _sid,PV-FUCP_*, *A _back,PV_*, *A _back,PV-UCP_*, *A _back,PV-FUCP_* are the area of the PV, PV-UCP and PV-FUCP side and back surface, respectively; *ε_1_*, *ε_2_*, *ε_3_*, *ε_4_*, *ε_5_*, are the optical emission of the top, side and back surface of PV. *ε_4_*, are optical emission of the side surface of the UCP. *T_0_*, is the room temperature; *T_1_*, *T_2_*, *T_3_*, *T_4_*, *T_5_*, *T_6_*, *T_7_*, *T_8_*, *T_9_*, are the temperature of top, side and back temperature of PV, PV-UCP and PV-FUCP, respectively.

**(3) Convention loss**

The convention loss of the pristine PV ($P_{conv,PV}$), PV-UCP ($P_{conv,PV-UCP}$), and PV-FUCP ($P_{conv,PV-FUCP}$) can be calculated by equation (5), (6) and (7), respectively,

| $P_{conv, PV}=A_{pro}h\left( T_{1}-T_{0} \right)+A_{sid,PV}h\left( T_{2}-T_{0} \right)+A_{back,PV}h\left( T_{3}-T_{0} \right)$ | (5) |
| --- | --- |
| $P_{conv, PV-UCP}=A_{pro}h\left( T_{4}-T_{0} \right)+A_{sid,PV-UCP}h\left( T_{5}-T_{0} \right)+A_{back,PV-UCP}h\left( T_{6}-T_{0} \right)$ | (6) |
| $P_{conv, PV-FUCP}=A_{pro}h\left( T_{7}-T_{0} \right)+A_{sid,PV-FUCP}h\left( T_{8}-T_{0} \right)+A_{back,PV-FUCP}h\left( T_{9}-T_{0} \right)$ | (7) |

where h is the convection heat transfer coefficient.

**(4) Latent heat**

The latent heat induced by the evaporation can be calculated by equation (8):

| $P_{latent}=M_{eva}H_{vap}A_{pro}$ | (8) |
| --- | --- |

where *M_eva_* is the evaporation rate, *H_vap_* is the equivalent evaporation enthalpy.

**Supplementary Figures**


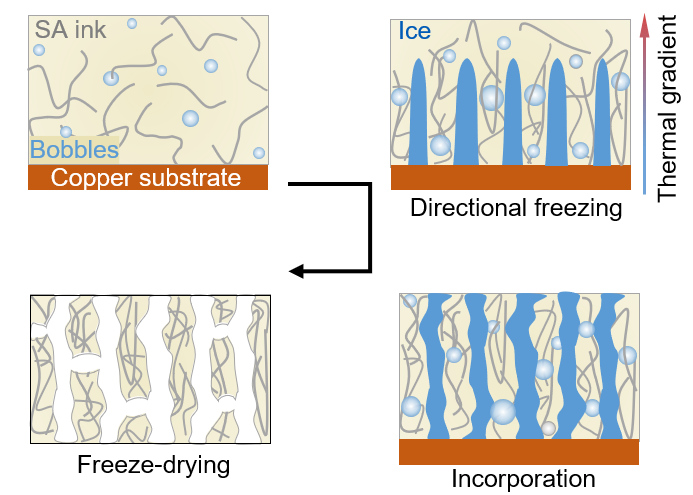


Figure S1. Preparation of the SA hydrogel skeleton. Aligned channels were achieved by directional freezing casting. The bubbles within the ink were interconnected with the channels to facilitate rapid mass transfer.


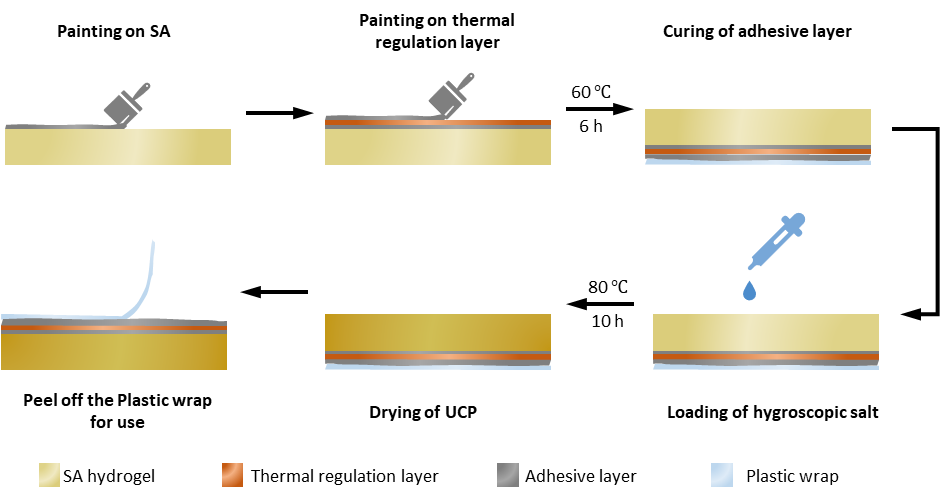


Figure S2. Preparation process of the UCP.


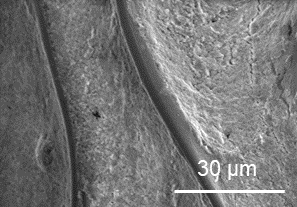


Figure S3. SEM of the AWH. The hygroscopic salts were uniformly distributed on the SA skeleton.


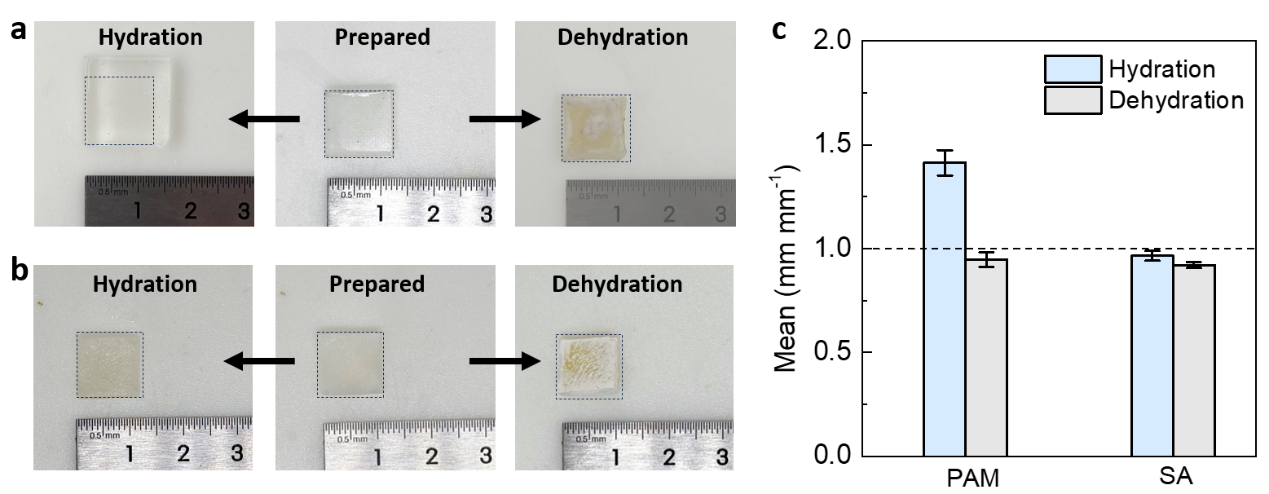


Figure S4. Comparison of the swelling ratios of SA and PAM hydrogels before and after water adsorption. Optical images of (a) PAM and (b) SA hydrogels in different states. (c) Swelling ratios of PAM and SA hydrogels in hydrated and dehydrated states.

The swelling ratio was defined as the length of the hydrated/dehydrated sample divided by the length of the prepared sample. The PAM exhibited an expansion of over 41% after hydration, whereas the size of the SA changed by only 3.4%. After dehydration, the PAM shrank significantly, whereas the size of the SA remained similar to that of the hydrated sample.


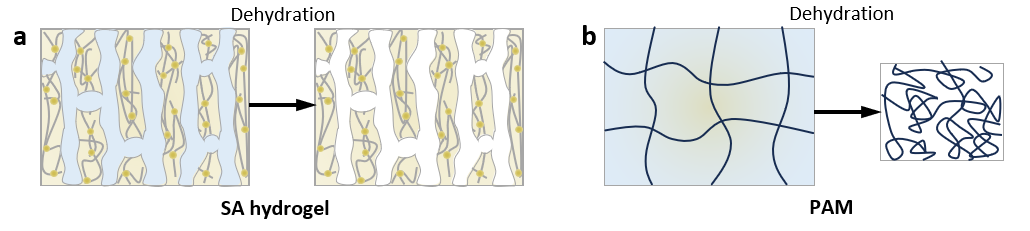


Figure S5. Schematic diagram of dehydration for (a) SA hydrogel and (b) PAM hydrogel. For the prepared SA hydrogel, during dehydration, water evaporates directly from the pores, and the rigid pore structure maintains its original shape without collapsing, similar to the dehydration process of a sponge (Figure S5a). In contrast, for the PAM hydrogel, when it absorbs water, the polymer chains swell due to the presence of hydrophilic groups, forming a three-dimensional network. During dehydration, as water is lost, the polymer chains contract and entangle, leading to pore structure collapse and significant volume reduction (Figure S5b).


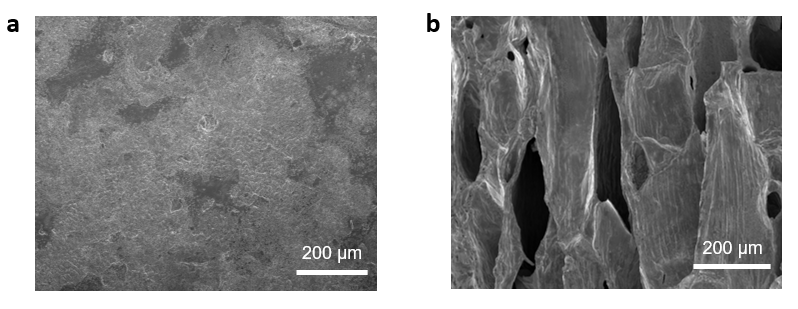


Figure S6. SEM image of the dehydrated (a) PAM and (b) SA hydrogel.

The fully dehydrated PAM exhibited a dense glass-like surface structure with no observable pores. This glassy surface creates significant mass transfer resistance and hinders the internal hygroscopic sites from interacting with vapor, thereby drastically reducing the moisture sorption performance (Figure S6). In contrast, the SA hydrogel maintains its porous structure even after complete dehydration. This porous structure results in a low mass transfer resistance and a large interface between air and the hygroscopic sites, facilitating rapid moisture capture and release.


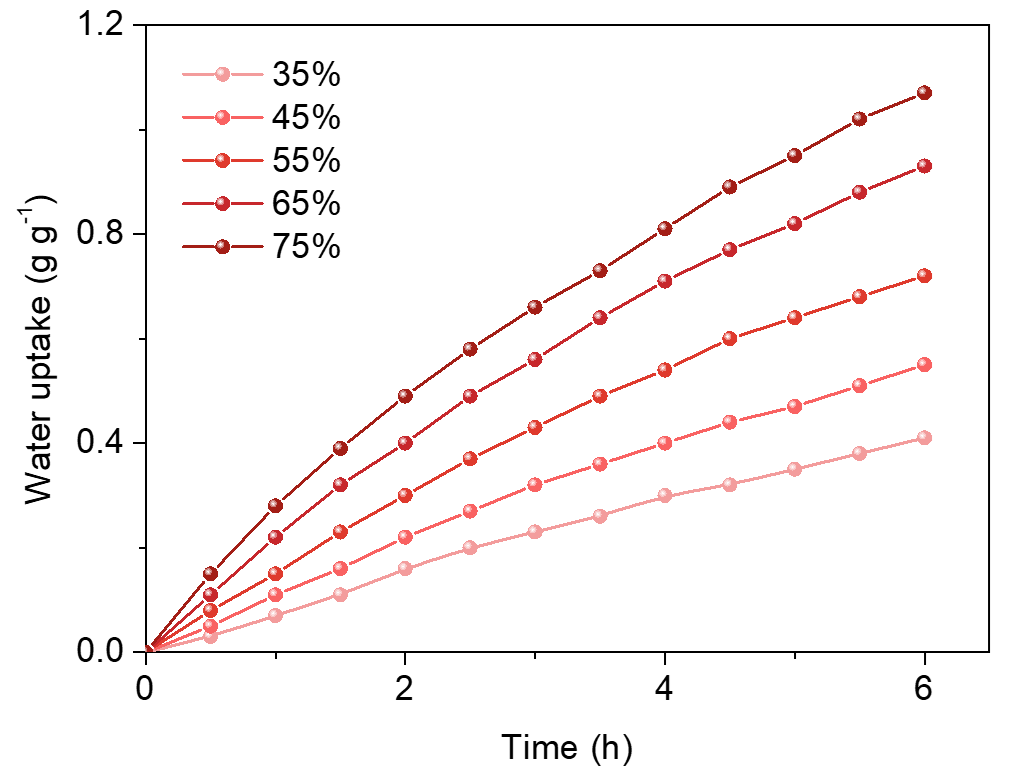


Figure S7. Water uptake performance of AWH under the different humidities.


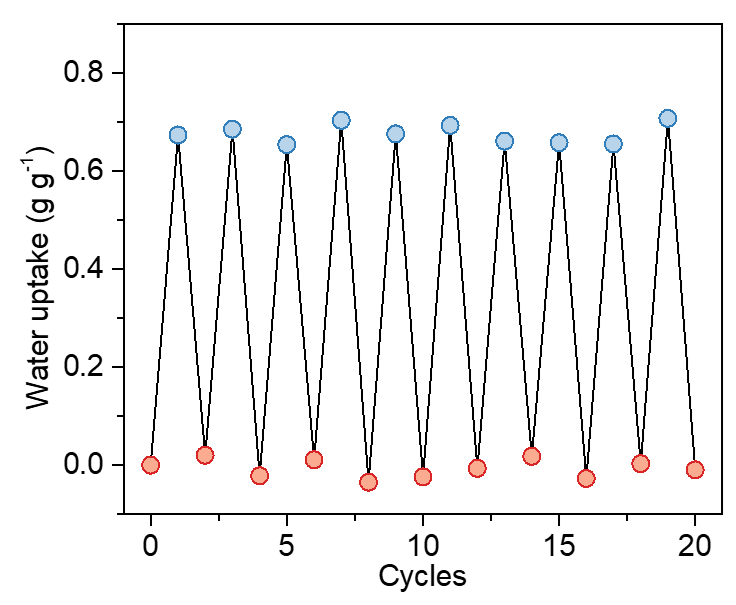


Figure S8. Water uptake stability of AWH during repeated adsorption-desorption cycles under 25 °C at 40% RH for sorption and 80 °C at 10% RH for desorption.


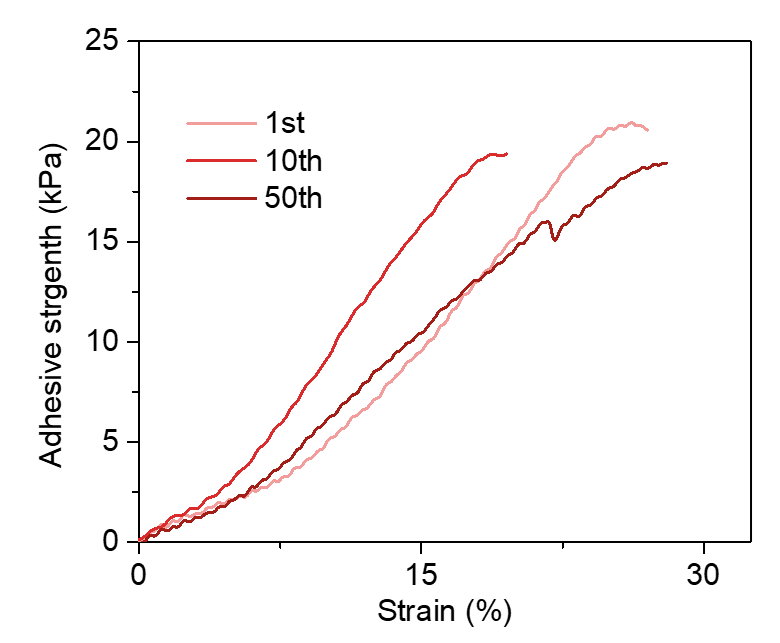


Figure S9. Lap-shear adhesion tests of UCP under repeated adhesion and detachment cycles on the silicon wafer.


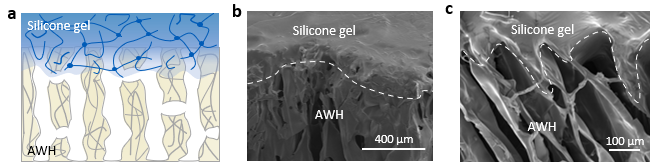


Figure S10. Adhesion mechanism between the silicone gel and cooling layer. (a) Interpenetrating polymer networks at the interface. (b-c) SEM images of the bonded interface.


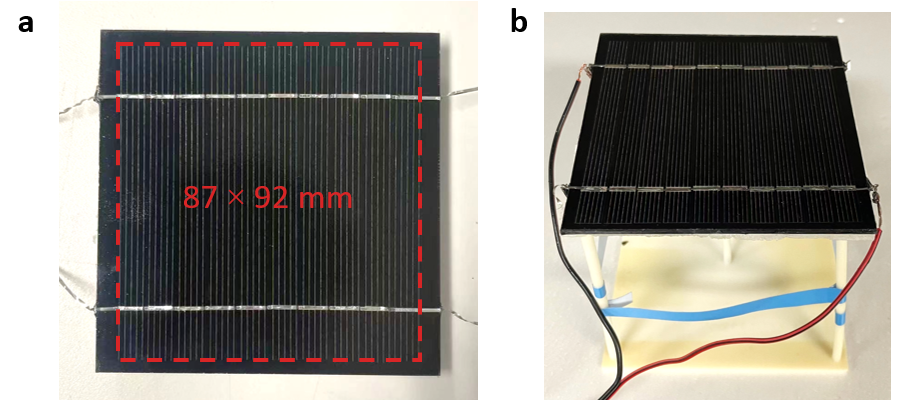


Figure S11. Test platform for cooling performance. (a) The effective dimensions of the PV panel are 87◊92 mm. (b) The PV or PV-UCP was positioned on the 3D printed scaffold for tests.


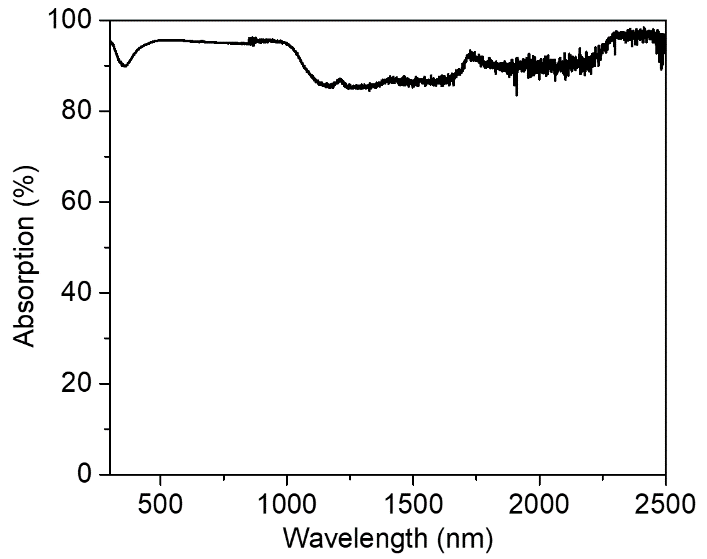


Figure S12. UV–Vis–NIR spectrum of the PV panel.


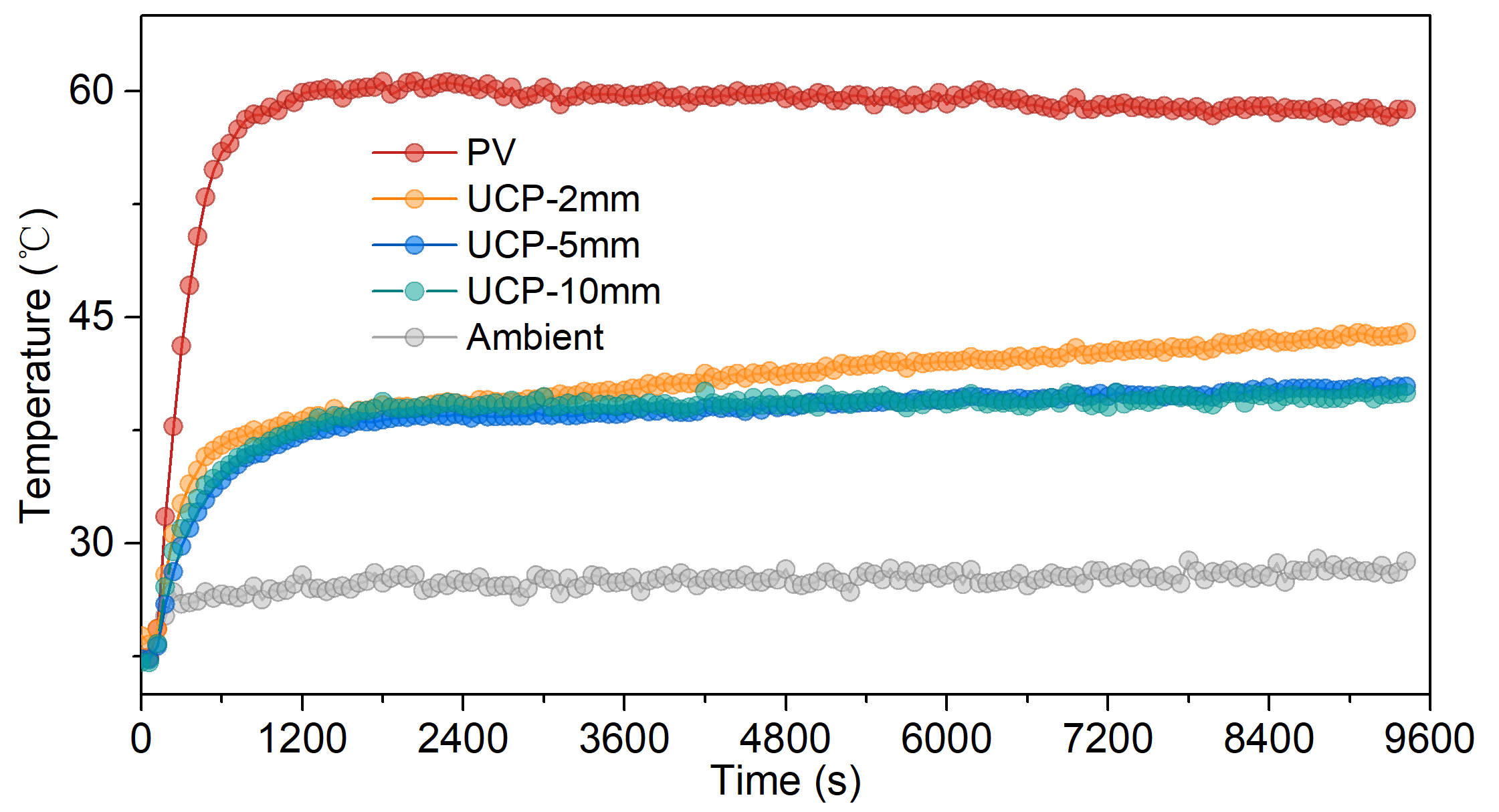


Figure S13. The effect of thickness of the AWH layer on the cooling performance. The pristine PV panel exhibited significantly higher temperatures when compared to the PV-UCPs, demonstrating the effective passive cooling of the UCP. During the first hour under 1 sun of illumination, the 3 PV-UCPs exhibited similar cooling performance. However, as the exposure time increased, the temperature of the PV-UCP with a 2 mm thickness slightly increased, while the temperatures of the PV-UCPs with 5 mm and 10 mm thicknesses remained constant. This is attributed to the thicker UCPs providing greater latent cooling capacity. As the evaporation progressed, the water content in the 2 mm thick PV-UCP rapidly declined, leading to its inferior cooling performance. In contrast, the UCPs with 5 mm and 10 mm thicknesses had sufficient water content, enabling them to sustain stable cooling performance.


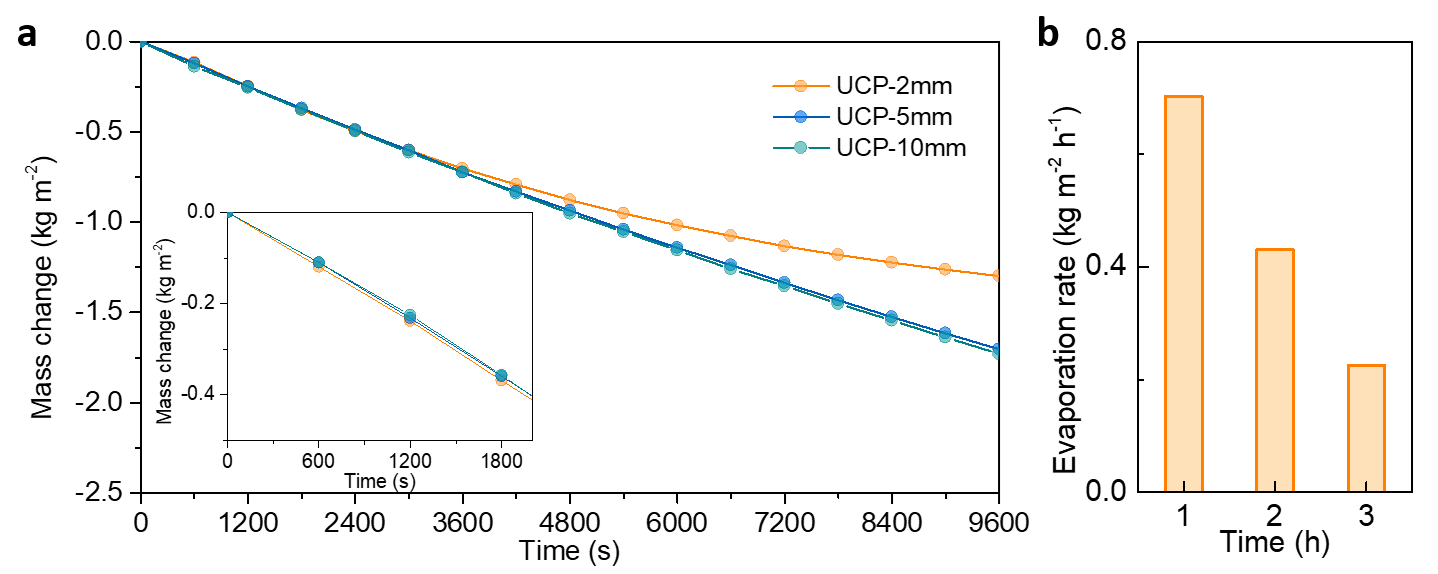


Figure S14. Evaporation performance of PV-UCP at various thicknesses. (a) Mass change of the PV-UCP with different thicknesses. The inset shows a zoomed-in view of the mass change during the first 30 min. (b) Changes in the evaporation rate of the PV-UCP with a thickness of 2 mm.

In the first 20 min, the evaporation rate of the 2 mm thick PV-UCP was slightly higher than those of the 5 mm and 10 mm (Figure S14). This was attributed to better heat transfer at lower thicknesses, which promoted evaporation. As the water content of the 2 mm thick system decreased, its evaporation rate dropped from 0.7 kg m² h⁻¹ to approximately 0.22 kg m² h⁻¹, affecting its cooling efficiency and leading to a temperature rise. In contrast, the 5 mm and 10 mm thick systems maintained a stable cooling performance owing to their adequate water storage.


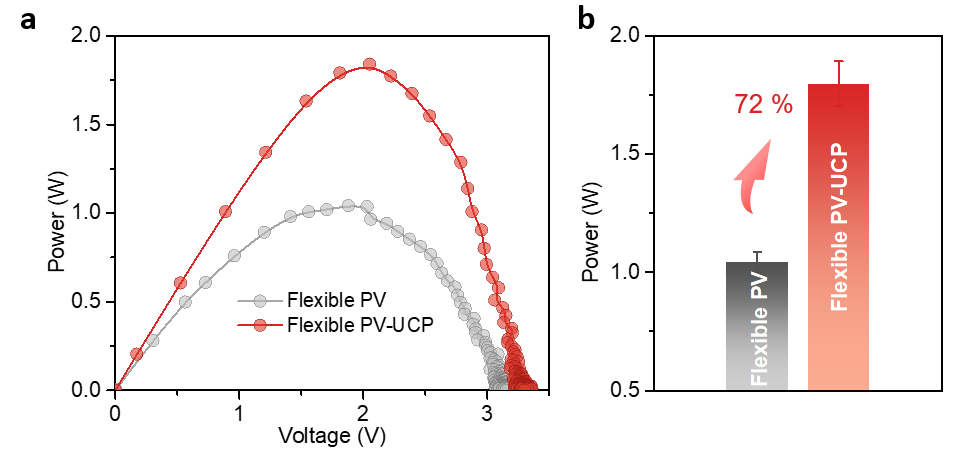


Figure S15. The power generation performance of flexible PV and flexible PV-UCP. (a) The power-voltage curves of pristine flexible PV and flexible PV-UCP. (b) In comparison of the maximum power density of the flexible PV and flexible PV-UCP, the maximum power density of the flexible PV-UCP was enhanced by around 72% due to the cooling effect of the UCP.

Figure S16. Global quarterly temperature distribution from 1850 to 2015.^1^


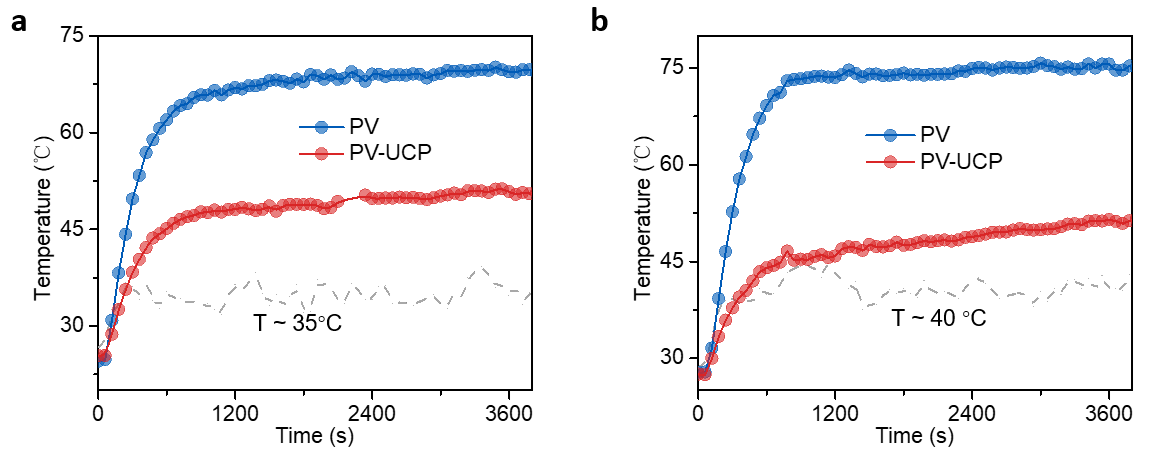


Figure S17. Temperature curves of the pristine PV panel and PV-UCP under 1 sun illumination at an ambient temperature of around 35 ℃ (a) and 40 ℃ (b), respectively.


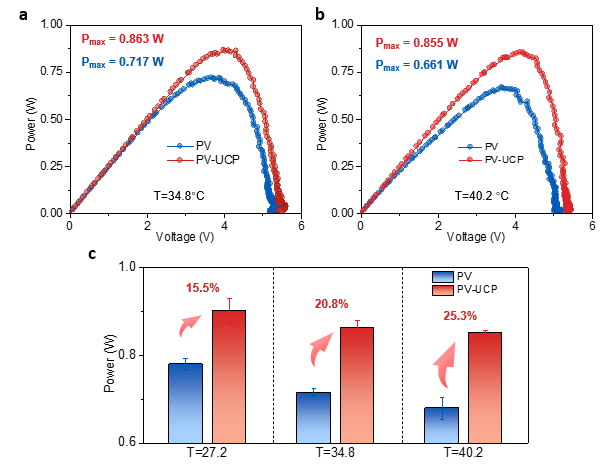


Figure S18. Power generation of PV-UCP at different temperatures. Power-voltage curves of PV and PV-UCP under 1 sun illumination at ambient temperature of around 35 ℃ (a) and 40 ℃ (b), respectively. (c) Power density comparison of the pristine PV and PV-UCP at different ambient temperatures, with the PV-UCP exhibiting a more pronounced effect as the temperature increases. The maximum power density of the pristine PV panel significantly declined from 0.717 W to 0.661 W as the ambient temperature increased from 35 ℃ to 40 ℃. In contrast, the maximum power density of the PV-UCP decreased by only 0.008 W, demonstrating the effective cooling performance provided by the UCP. This simultaneously proves that the passive cooling strategies employed by the UCP are still efficient even under high-temperature operating conditions.


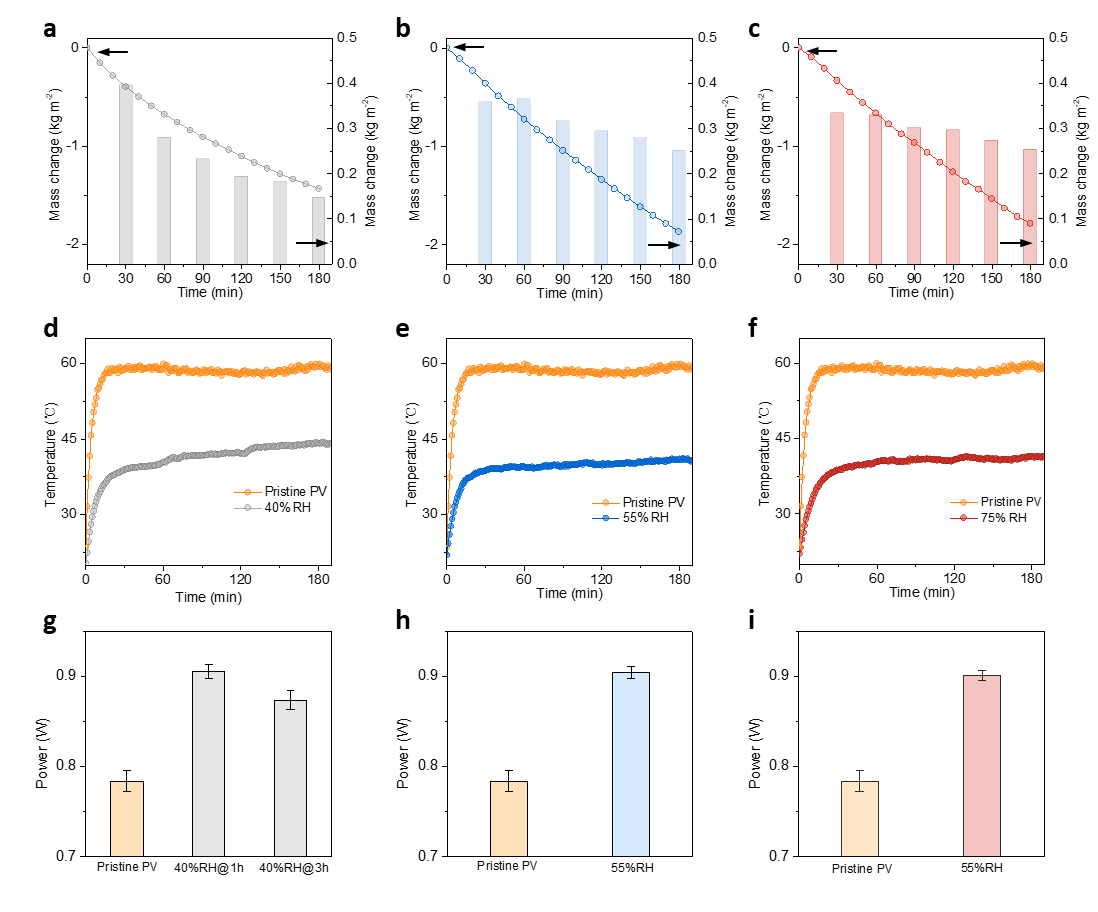


Figure S19. The impact of humidity on the cooling performance of UCP. (a-c) Mass change, (d-e) temperature, and (g-i) power density of PV-UCP at humidities of 40% RH, 55% RH, and 70% RH, respectively.

Humidity is critically important for cooling performance and primarily affects two key aspects: the atmospheric water harvesting capacity and evaporation rate. First, regarding atmospheric water harvesting capacity, humidity determines the vapor sorption speed and the amount of stored water in the cooling layer, which is vital for its sustained cooling effectiveness. At low humidity, a significant drop in sorption can significantly reduce the effective duration of the cooling layer, thus negatively affecting the cooling performance. Second, concerning the evaporation capacity, low humidity promotes cooling by creating a larger vapor pressure difference at the evaporation interface, accelerating evaporation, whereas high humidity suppresses evaporation and hinders the cooling effect.

To evaluate the cooling performance of UCP at different humidity levels, three sets of PV-UCP were placed under conditions of 40% RH, 55% RH, and 75% RH for 12 h of sorption, followed by exposure to 1 sun of solar irradiation at the corresponding humidities. In the first 30 min of testing, the mass change at 40% RH was 0.12 kg m^-2^ higher than that at 75% RH (Figure S19a). This is because of the lower humidity, which creates a larger vapor pressure difference, which is more favorable for evaporation. However, as the test progressed, the evaporation rate at 40% RH significantly decreased compared to 55% RH and 75% RH, dropping from 0.396 kg m^-2^ to 0.147 kg m^-2^within 3 h (Figure S19b-c). This decline occurred because the water storage capacity of UCP at 40% RH was insufficient to sustain continuous evaporation. The PV temperature tests further demonstrated the effect of humidity on the cooling performance of the UCP. Under 40% RH conditions, the PV temperature gradually increased from 39.6 °C to around 44.5 °C (Figure S19d). This increase in temperature was attributed to the limited water storage capacity of the UCP at low humidity, which reduced its cooling effectiveness during prolonged operation. In contrast, the photovoltaic temperatures at 55% RH and 75% RH remained relatively stable, at 40.7 and 41.5 degrees, respectively (Figure S19e-f). The slightly lower temperature at 55% RH was due to the favorable evaporation conditions, which enhanced the cooling effect of UCP. The power-generation performance was recorded, as shown in Figure S19g-i. The pristine PV exhibited a low power density of 0.783 W, whereas the PV-UCP at 40% RH showed an initial power density of 0.905 W, which slightly decreased to 0.873 W after 3 h. The PV-UCP at 55% RH and 75% RH demonstrated relatively stable power densities of 0.904 W and 0.901 W, respectively.

Based on the above results, humidity has a significant impact on the water absorption capacity of the UCP, which notably affects the long-term stability of its cooling performance. Theoretically, humidity also influences the evaporation rate, thereby affecting the power performance. However, this effect was not pronounced in the present study. By adjusting the humidity from 75% RH to 40% RH, the power density decreased by approximately 0.5%. Previous studies have shown similar results; for instance, increasing humidity from 40% to 80% resulted in a temperature change of about 1.5 °C and a power impact of approximately 0.8%.^2^ This may be due to the limited humidity range tested, as extreme humidity (particularly below 30% RH) would likely have a more significant effect. However, this is difficult to achieve and maintain under current experimental conditions. Future research should focus on sorption-based PV cooling under extreme humidity conditions.


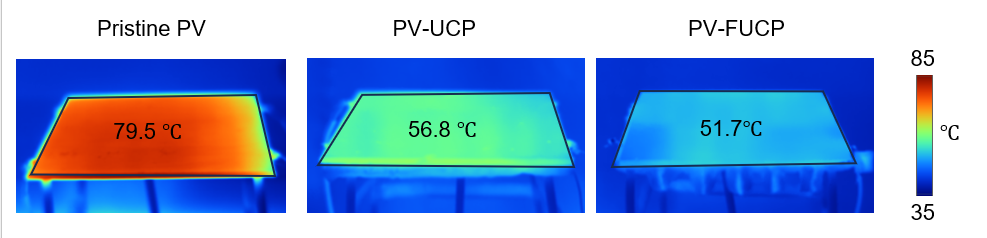


Figure S20. IR images of Pristine PV, PV-UCP, and PV-FUCP under 1 sun. The slight difference between the IR image and the thermocouple may be due to the positioning of the thermocouple during testing.


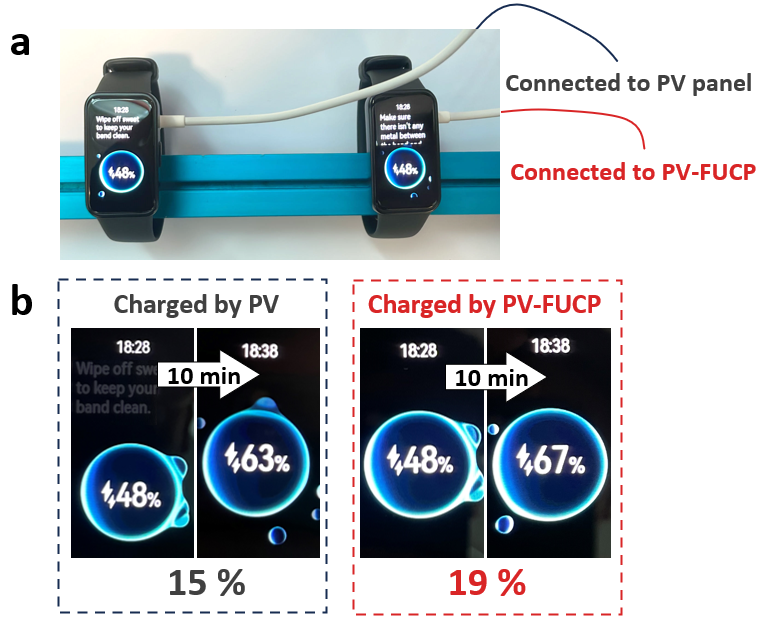


Figure S21. Comparison of the charging capacity for a smartwatch using pristine PV panel versus PV-FUCP under 1 sun illumination for 10 min. (a) Setup for charging the identical smartwatch (Huawei Band 8) using a PV panel and PV-FUCP. (b) The increase in battery charge of the smartwatches after 10 min of charging.


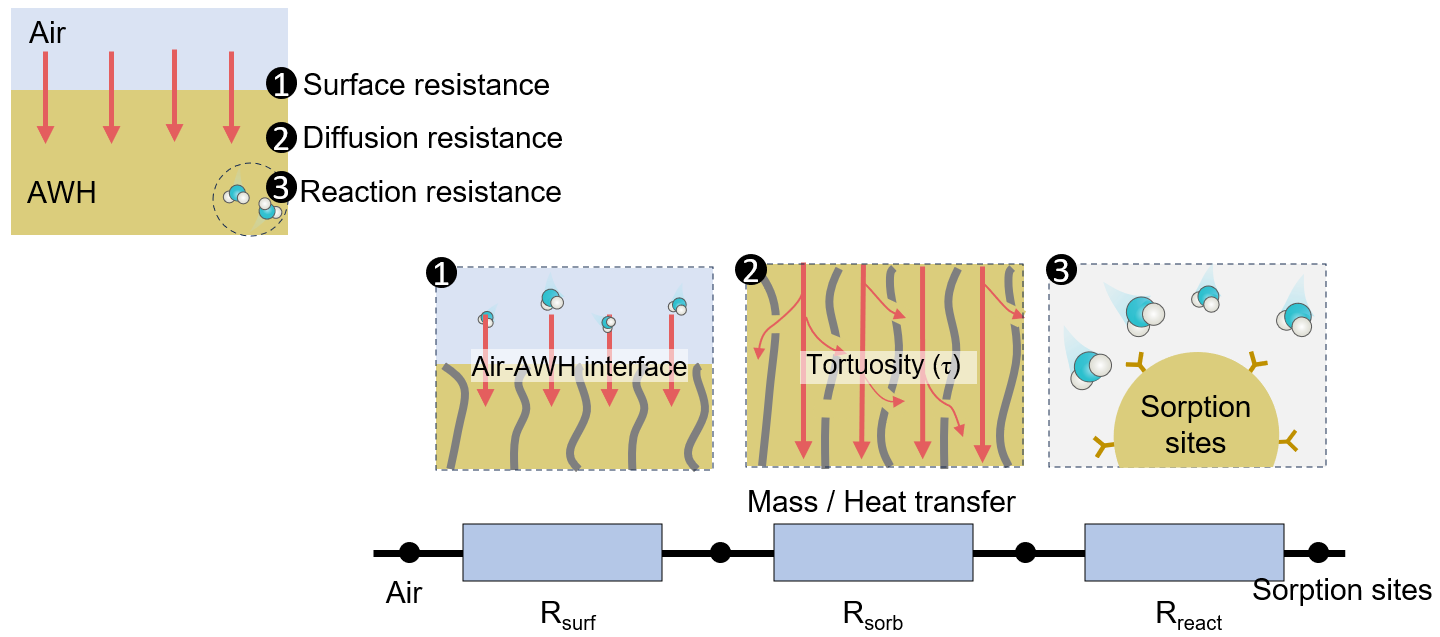


Figure S22. Schematic of fast mass/heat transport in AWH.

There are three steps in moisture capture: i) Water molecule transport from the air to the AWH surface. ii) Water molecule diffusion within the AWH through the oriented microchannels and micropores. iii) Water molecules were captured by the sorption sites of the sorbents. During the multistep sorption process, the heat- and mass-transport efficiencies were mainly determined by the three transport resistances.

Surface resistance

$$R_{surf}=\frac{1}{h}\cdot\frac{A_{ext}}{A}$$

Diffusion resistance

$$R_{sorb}=\frac{\tau}{\varepsilon}\cdot\frac{\delta_{sorb}}{D_{p}}\cdot\frac{A_{ext}}{A}$$

And reaction resistance

$$R_{react}=\frac{1}{K_{r}}\cdot\frac{A_{ext}}{V}$$

where A represents the interfacial area between the nanocomposite and air. A_ext_ is the external surface area of the nanocomposite. where h is the surface transport coefficient, which is influenced by the airflow rate. δ_sorb_ is the transport depth. D_p_ is the diffusivity of water vapor in the pore, ε is the porosity, and τ represents the tortuosity. K_r_ represents the reaction rate coefficient which can be expressed as K_r_=1/τ_r_, τ_r_ is the characteristic reaction time. The key research objective for fast moisture capture is to lower diffusion resistance, which can be tuned by the diffusion depth and tortuosity. The low tortuosity of the prepared AWH enabled low mass transfer resistance, leading to fast sorption/evaporation kinetics.


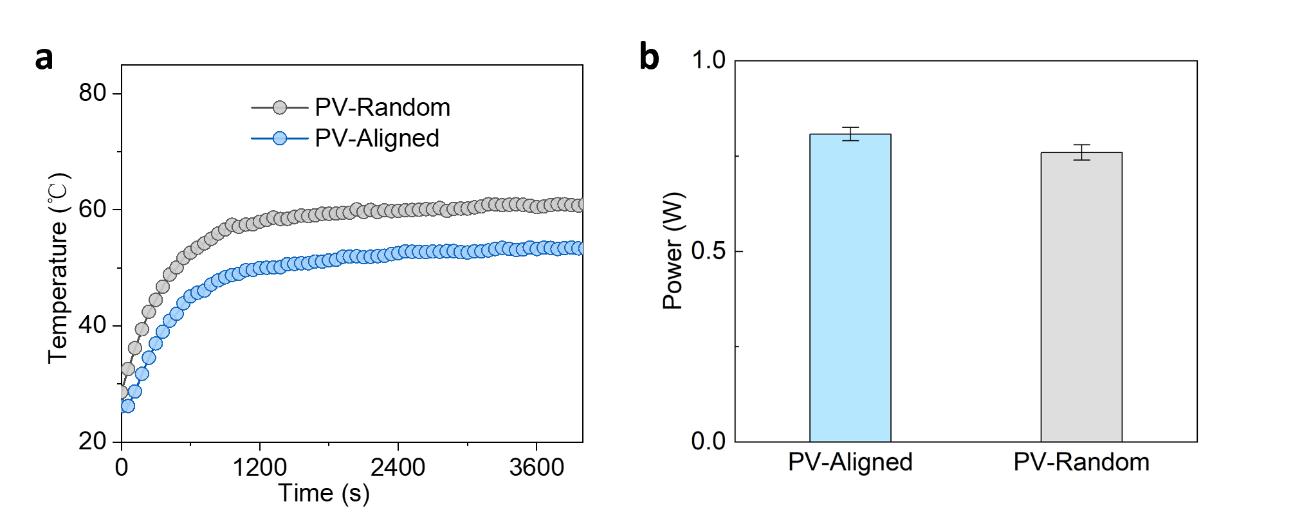


Figure S23. Comparison of cooling performance between the SA with random channels and the SA with aligned channels. (a) Temperature and (b) power generation of the PV with different SA.

The temperature of the PV panel with the random channel increased by approximately 7 ℃, while the power generation performance decreased by 0.048 W (Figure S23). The cooling power of the SA with the random channel was calculated to be 469 W m^-^², which is lower than that of the SA with the aligned channel (using surface area rather than projected area for calculation). This result highlights the advantages of the aligned channel.


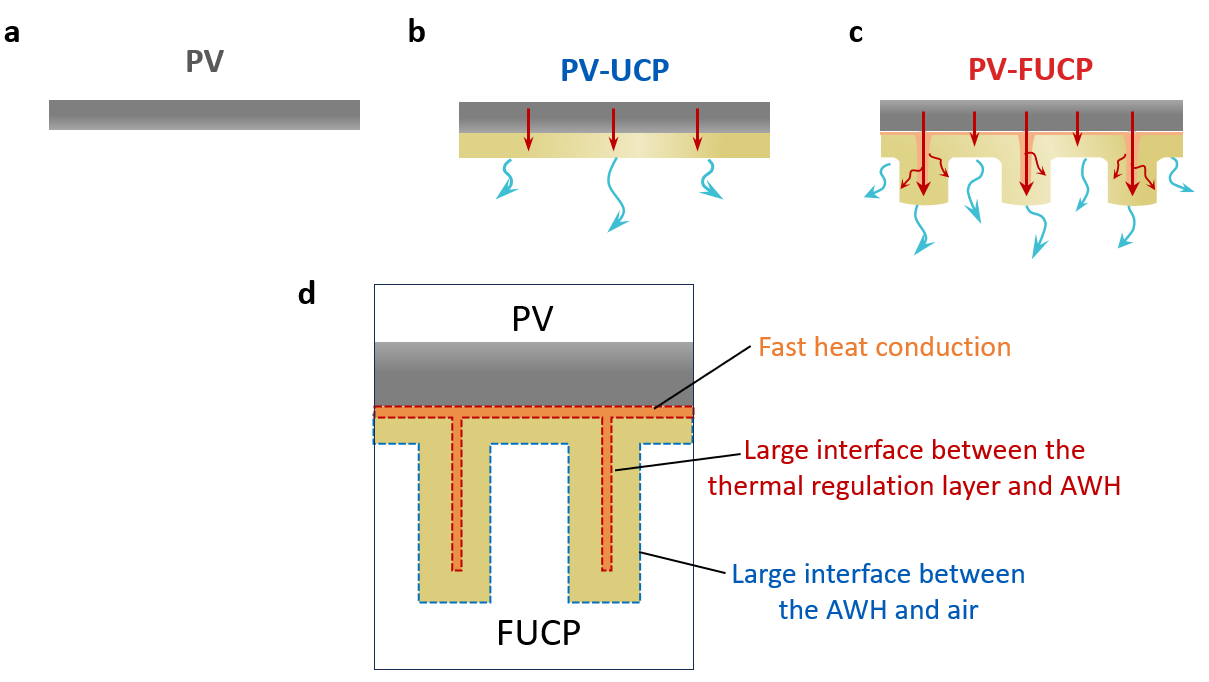


Figure S24. Heat conduction and evaporation cooling in (a) pristine PV, (b) PV-UCP, and (c) PV-FUCP systems, respectively. (d) The high conductivity of the thermal regulation layer (TRL, 94 W (m·K)^-1^) and the large TRL-AWH interface enables fast waste heat transfer from the PV panel to the FUCP, while the large AWH-air interface facilitates vapor escape for ultra evaporation cooling.


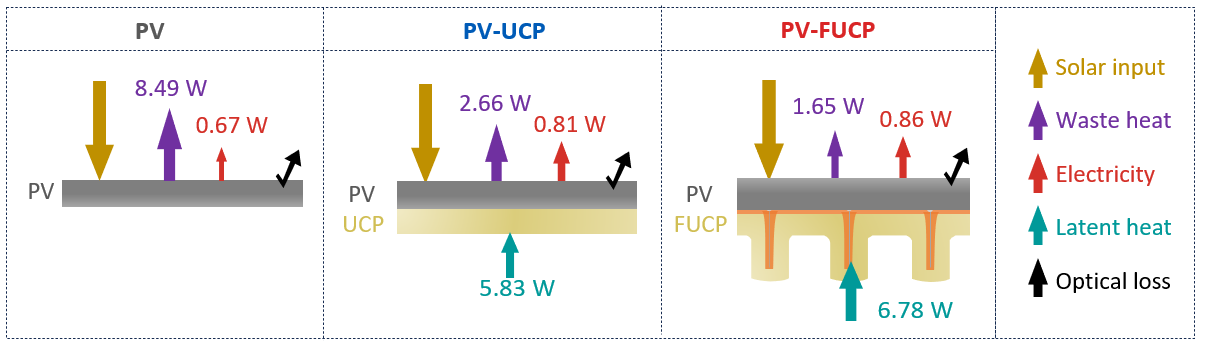


Figure S25. Calculated energy flow of the pristine PV, PV-UCP and PV-FUCP. For the pristine PV panel, over 80% of the incident solar energy was converted to heat (i.e., 8.49W), which was then dissipated into the environment. Whereas the PV-UCP removed a significant amount of heat (5.83W) by water evaporation, accounting for 69% of the total waste heat. After reshaping the UCP, the cooling performance was passively enhanced, leading to an increase in the latent heat by 0.95W and an ultra-high cooling power of 692 W m^-2^. The PV system was suspended on a 3D-printed scaffold (Figure S11), and the conductive heat loss from the support can be considered negligible.


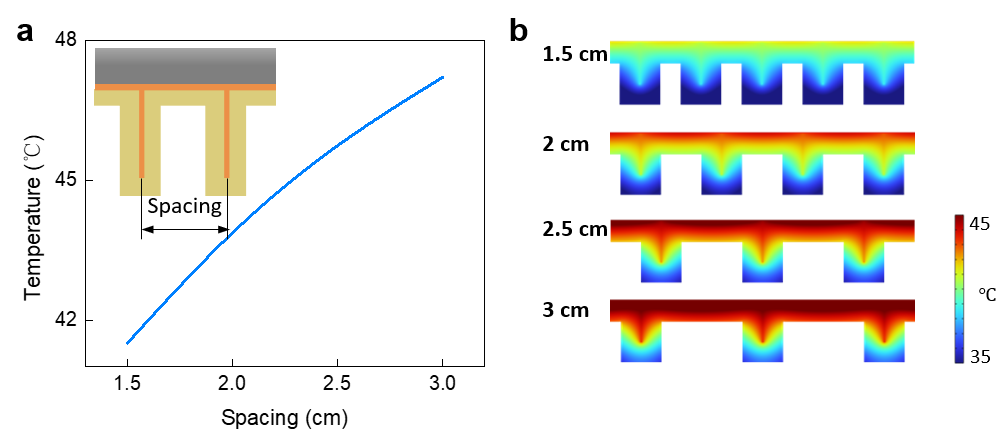


Figure S26. Effect of spacing distance on the cooling performance. (a) Average temperature of PV with different spacing distances. (b) Simulated temperature of PV-FUCP. An increase in fin spacing results in a reduction in the total number of heat-dissipation fins, consequently decreasing the cooling performance and raising the operational temperature of the PV panel.


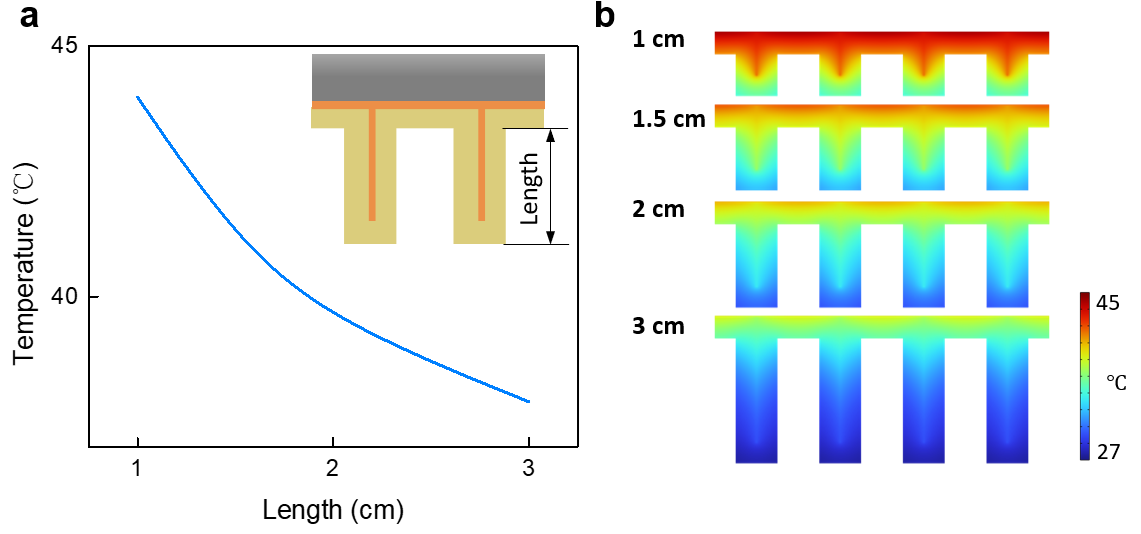


Figure S27. Effect of length of the folded area on the cooling performance. (a) Average temperature of PV with different lengths. (b) Simulated temperature of PV-FUCP with different lengths. The average temperature of the PV panel decreased notably with an increase in the length of the fin. The longer fins provided greater interfacial area between the thermal regulating layer and AWH, as well as between the air and AWH. This facilitated enhanced water evaporation, thereby cooling down the PV panel.


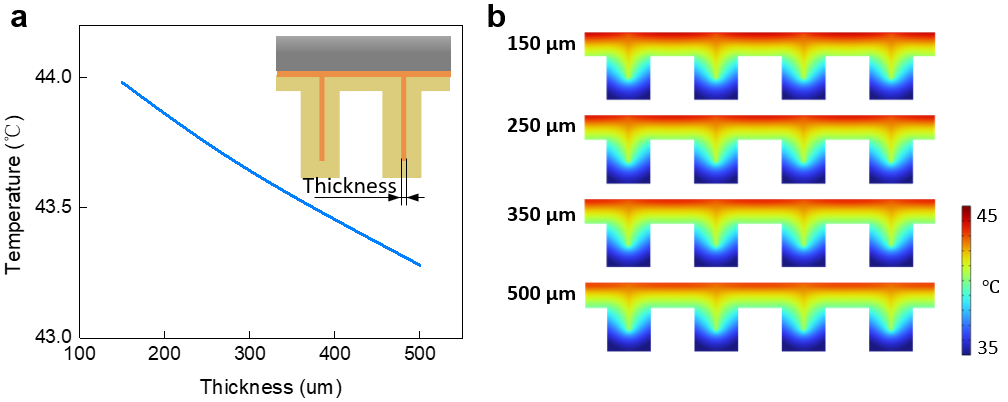


Figure S28. Effect of thickness of the thermal regulating layer on the cooling performance. (a) Average temperature of PV with different thicknesses of the thermal regulating layer. (b) Simulated temperature of PV-FUCP with different thicknesses of the thermal regulating layer.


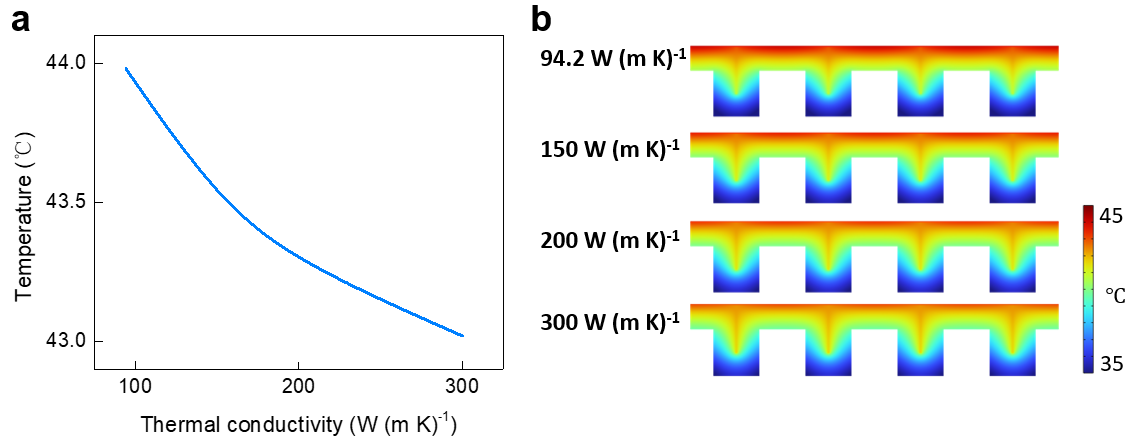


Figure S29. Effect of the thermal conductivity of the thermal regulating layer on the cooling performance. (a) Average temperature of PV with different thermal conductivities of the thermal regulating layer. (b) Simulated temperature of PV-FUCP with the different thermal conductivities of the thermal regulating layer.

Augmenting the thickness and thermal conductivity of the thermal-regulating layer can enhance the heat-transfer capacity of the FUCP, which promotes heat dissipation and results in a lower temperature of the photovoltaic panel temperature.


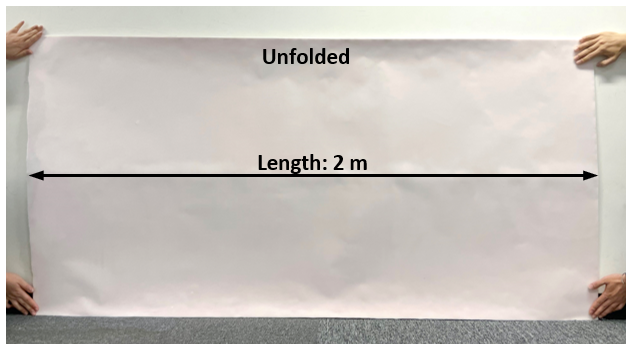


Figure S30. Optical image of large-scale UCP with size of 2000 mm x 1000 mm.


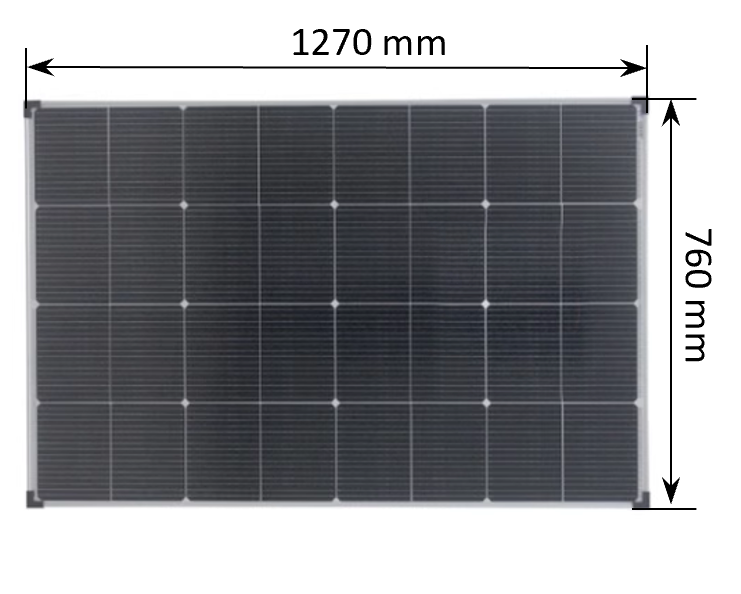


Figure S31. The commercial PV panel. The area is around 1 m^2^.


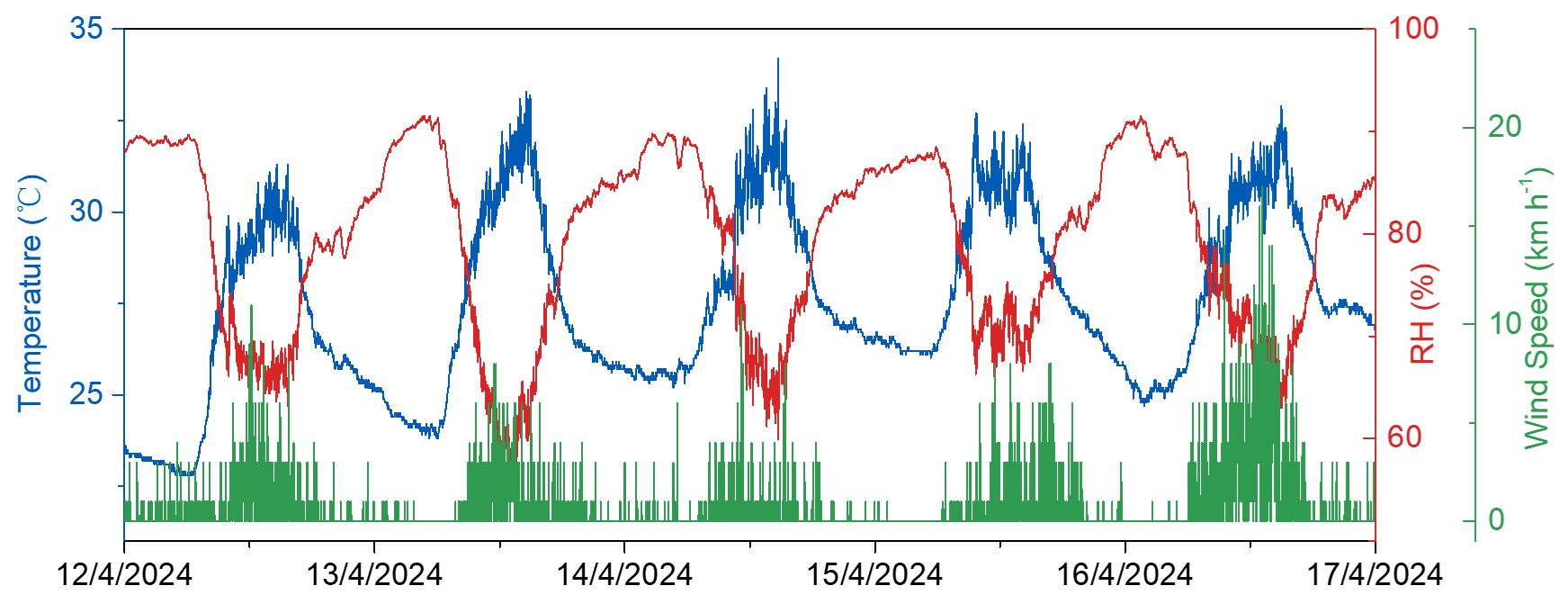


Figure S32. The temperature, humidity and wind speed during the outdoor test.


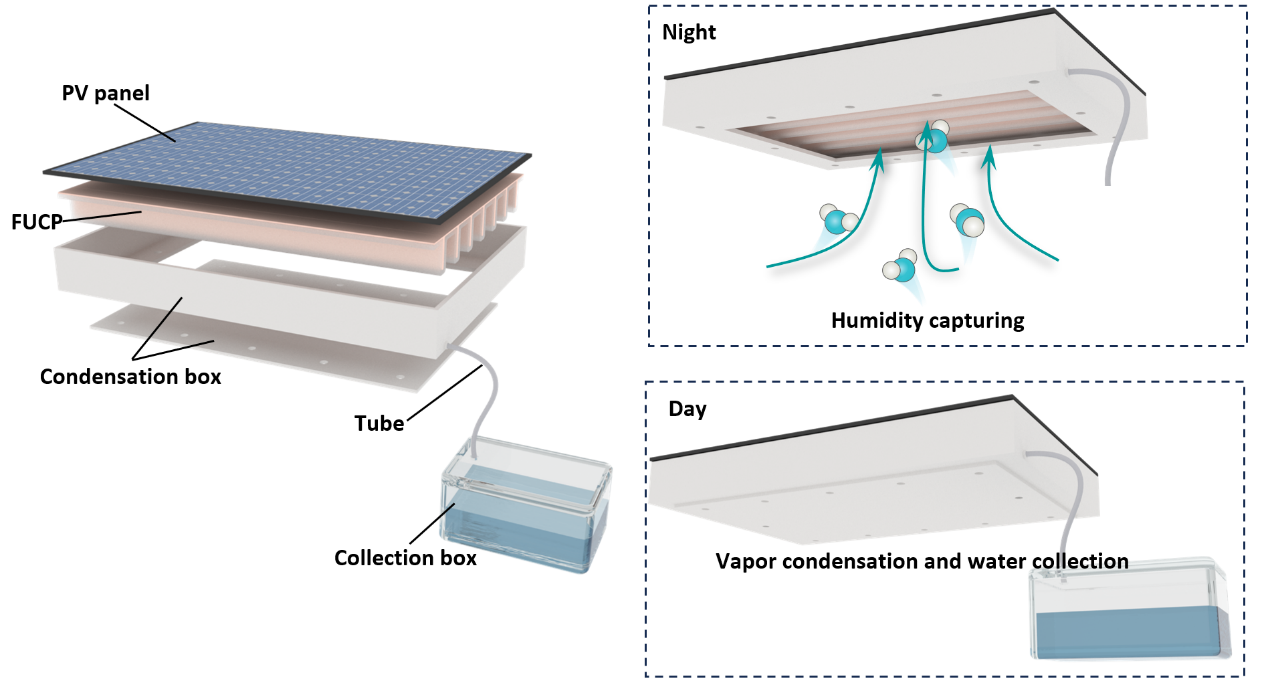


Figure S33. The designed water condensation chamber. The condensation box was fixed behind the PV panel, a tube was connected to the condensation chamber for water collection. During the nighttime, the rear panel of the condensation chamber was opened to facilitate humidity capture. Conversely, during the daytime, the rear panel of the condensation chamber was closed to enable vapor condensation and water collection.

**Table S1. Comparison of UCP with previously reported sticky cooling patches.**

| **Ref No.** | **Temperature drop (℃)** | **Power enhancement (%)** | **Testing condition** | | | **Features** |
| --- | --- | --- | --- | --- | --- | --- |
|  |  |  | **Humidity (%)** | **Temperature (℃)** | **Stability (h)** |  |
| ^3^ | 7.5 | 2.4 | / | / | 6^a^ | Permanent adhesion |
| ^4^ | 9.14 | 4.8 | / | ~ 25 | / | Adhered to the top surface of PV |
| ^2^ | 13 | / | 20-80 | 10-40 | 1.5 | Adhesion |
| ^5^ | 11.8 | 10.2 | 90 | 25 | 10^b^ | Adhesion |
| ^6^ | 5 | / | / | / | / | Adhesion |
| ^7^ | 20 | / | 60 ^c^ | 25 | 6 ^d^ | Adhesion |
| This work | 29.1 | 28 | 40-75 | 25-40 | 5 | Adhesion / regulating heat transfer path |

^a^ The cooling layer was tested on a heat plate at nearly 38 °C above ambient temperature, and after 6 h of continuous operation, its temperature closely matched that of the heat plate; ^b^ The light intensity varied from 0 to 1 sun over 10 hours, with a 1 m/s airflow to ensure sufficient moisture capture; ^c^ Water capture was conducted at 60% RH humidity for 20 h; ^d^After 6 h of conduction, the cooling performance decreased from 20 °C to 15 °C.

**Table S2.** **Comparison on the cooling performance of this work with 20 previous reported reprehensive references.**

| **Item No.** | **Mechanism** | **Temperature drop (℃)** | **Cooling power (W m^-2^)** | **Operating range (℃)** | **Materials / Strategies** | **Comments** | **Year** | **Journal** | **Ref** |
| --- | --- | --- | --- | --- | --- | --- | --- | --- | --- |
| This work | Evaporation cooling | 29.1 | 692 | ~40 | Self-prepared ultra cooling patch | Engineering-simple, user-friendly and scalable | 2024 | / | / |
| 1 | Radiative cooling | 8.8 | >130 | / | Hierarchically porous ceramics | / | 2023 | Science | ^8^ |
| 2 | Radiative cooling | >9°C during the night and >4°C during midday | 53 | 13-28 | Cooling wood | Average cooling power of 53 W m^-2^ over the 24-hour period | 2019 | Science | ^9^ |
| 3 | Radiative cooling | 5.1 | 63.8 | 18-24 | Radiative cooler & IR-opaque layer | 63.8 W m^-2^ under peak sunlight and 87.0 W m^-2^ at night | 2024 | Cell Reports Physical Science | ^10^ |
| 4 | Radiative cooling | 6 | / | / | Micro-grating integrated cooler | / | 2021 | Advanced Energy Materials | ^11^ |
| 5 | Radiative cooling | 36 | 310 | / | Cooling assembly system | Concentrating photovoltaics | 2020 | Joule | ^12^ |
| 6 | Radiative cooling | 4.7 | 92 | / | Photonic cooler | / | 2020 | Optics Express | ^13^ |
| 7 | Radiative cooling | 17 | 200 | 20 | Random inverted pyramid textured PDMS | / | 2022 | Solar Energy | ^14^ |
| 8 | Radiative cooling | 5.7 | 149 | 40–70 °C | Multilayer photonic film (Al2O3/SiN/TiO2/SiO2) | / | 2021 | ACS Photonics | ^15^ |
| 9 | Radiative cooling | 13 | 96 | ~25 | Custom-fabricated polyethylene aerogel | / | 2019 | Science Advances | ^16^ |
| 10 | Evaporation cooling | 5-10 | 298 | / | Carrageenan, lithium chloride (LiCl) and polypyrrole (PPy) | / | 2022 | Journal of Materials Chemistry A | ^17^ |
| 11 | Evaporation cooling | 10 | 295 | 20-70 | PAM-CNT-CaCl2 | / | 2020 | Nature Sustainability | ^18^ |
| 12 | Evaporation cooling | 26 | 590 | 24.1 and 33.5 | potassium polyacrylate |  | 2023 | Nature Communications | ^19^ |
| 13 | Evaporation cooling | 10.6–12.6 | 68.9–136.1 | / | MOF coated heat sink |  | 2024 | Materials Advances | ^20^ |
| 14 | Evaporation cooling | 7.5 | / | / | CaCl_2_/PAM/SA hydrogel | Maximum temperature reduction at outdoor test | 2023 | ACS Energy Letters | ^3^ |
| 15 | Evaporation cooling | 23 | / | / | Self-prepared PU |  | 2024 | Nature Water | ^21^ |
| 16 | Evaporation cooling | 18 | / | 26 | PVA/CH phase change hydrogel and MOF@PVA/CH bilayer |  | 2023 | Nano Energy | ^22^ |
| 17 | Evaporation cooling | 11 | 367 | 25 | Thermal interconnecting layer & solar water purifier | Under 1 sun | 2020 | Joule | ^23^ |
| 18 | Salinity difference induced evaporation | 3 | 170 | 29-30 | Distilled water and brine |  | 2020 | Science Advances | ^24^ |
| 19 | Evaporation cooling & Radiative cooling | 7 °C sub-ambient temperature | 150 | 20-60 | P(VDF-HFP) film & Li-PAAm hydrogel | Sub-ambient temperature | 2021 | Nano Energy | ^25^ |
| 20 | Desorption-driven endothermic reaction | 15.1 | 403 | 20-70 | 13X/H_2_O/NH_4_NO_3_ |  | 2023 | Nature Communications | ^26^ |

**Table S3. Comparison on the cooling performance of this work with 6 representative works that employ evaporation layers for PV cooling**

| **Ref No.** | **Mechanism** | **Temperature drop (℃)** | **Cooling power (W m^-2^)** | **Testing condition** | |
| --- | --- | --- | --- | --- | --- |
|  |  |  |  | **Humidity (%)** | **Temperature (℃)** |
| ^19^ | Evaporation cooling | 26 | 590 | 10 | 33.5 |
| ^18^ | Sorption-evaporation cooling | 10 | 295 | 60 | 22 |
| ^26^ | Desorption-driven endothermic reaction | 15.1 | 403 | / | 20-70 |
| ^2^ | Sorption-evaporation cooling | 13 | 356 ^a^ | 20-80 | 10-40 |
| ^5^ | Sorption-evaporation cooling | 11.8 | 247 ^b^ | 90 | 25 |
| ^27^ | Sorption-evaporation cooling | 9.9 | 288.2 | 60 | 25 |
| This work | Sorption-evaporation cooling | 29.1 | 692 ^c^ | 40-75 | 25-40 |

^a^ The cooling power of 356 was measured at 60% RH, 40 ℃; ^b^ The cooling power of 356 was measured under light intensity of 725 W m^-^²; ^c^ The cooling power of 692 was measured at 55% RH, 40 ℃. The PV area was used to calculate the cooling power. The surface areas of the UCP and FUCP are 1.2 and 2.2 times larger than that of the PV panel, respectively. The calculated cooling power for the UCP and FUCP, based on the cooling layer’s surface area, is 538 W m^-2^ and 315 Wm^-2^ respectively. While the FUCP exhibits a lower cooling power density due to its larger surface area, it still provides superior overall cooling performance for the PV panel due to its enhanced heat dissipation capability.

**Table S4. Material costs of the UCP.**

| **Items** | **Uint cost (USD)** | **Cost (USD/each)** |
| --- | --- | --- |
| PDMS | 94.81 /kg | 0.068 |
| Cu | 8 /kg | 0.107 |
| Porous sponge | 20.6 /m^3^ | 0.01 |
| CaCl_2_ | 3.31/kg | 0.04 |
| Total | | 0.225 |

The material cost of each UCP was estimated to be approximately $0.225. Copper and PDMS account for 77.8% of the total cost. By replacing these materials with aluminum and silica gel, material costs can be significantly reduced. When integrated with a 200W PV panel, the enhancement in power generation performance is estimated to be around 56.94 kWh per year (with an average of 6.5 hours of sunlight per day). With a rooftop PV capacity of 10 kW (for example, in Hong Kong), the passive cooling system could generate approximately $18,320 in revenue over ten years, while the costs are approximately $1587, which includes the annual maintenance and material costs of the UCP. This analysis also highlights the application potential of UCP, particularly in Hong Kong.

**Supplementary References**

(1) Rohde, R. A.; Hausfather, Z. The Berkeley Earth Land/Ocean Temperature Record. *Earth Syst. Sci. Data* **2020**, *12* (4), 3469–3479. https://doi.org/10.5194/essd-12-3469-2020.

(2) Cai, J.; Li, W.; Jin, S.; Shen, L.; Wang, B.; Gan, Z.; Pan, Q.; Zheng, X. Thermal Management Performance Study of PV Adsorptive Evaporative Cooling Based on Noncorrosive Salt-Embedded Composites. *Renew. Energy* **2024**, *237* (PB), 121805. https://doi.org/10.1016/j.renene.2024.121805.

(3) Li, Z.; Ma, T.; Ji, F.; Shan, H.; Dai, Y.; Wang, R. A Hygroscopic Composite Backplate Enabling Passive Cooling of Photovoltaic Panels. *ACS Energy Lett.* **2023**, *8* (4), 1921–1928. https://doi.org/10.1021/acsenergylett.3c00196.

(4) Shang, J.; Zhang, J.; Zhang, Y.; Zhang, X.; An, Q. Highly Potent Transparent Passive Cooling Coating via Microphase-Separated Hydrogel Combining Radiative and Evaporative Cooling. *Nano Lett.* **2024**, *24* (23), 7055–7062. https://doi.org/10.1021/acs.nanolett.4c01621.

(5) Fang, H.; Dang, S.; Kumar, P.; Wang, J.; Xu, L.; Zhu, Y.; Almogbel, A.; Albadri, A.; De Wolf, S.; Gan, Q. Streamlined Fabrication of an Inexpensive Hygroscopic Composite for Low Maintenance Evaporative Cooling of Solar Panels. *Mater. Sci. Eng. R Reports* **2025**, *165* (April), 101016. https://doi.org/10.1016/j.mser.2025.101016.

(6) Lv, T.; Sun, L.; Yang, Y.; Huang, J. Bio-Inspired Hydrogel with All-Weather Adhesion, Cooling and Reusability Functions for Photovoltaic Panels. *Sol. Energy* **2021**, *216* (10), 358–364. https://doi.org/10.1016/j.solener.2021.01.028.

(7) Mu, X.; Shi, X.-L.; Zhou, J.; Chen, H.; Yang, T.; Wang, Y.; Miao, L.; Chen, Z.-G. Self-Hygroscopic and Smart Color-Changing Hydrogels as Coolers for Improving Energy Conversion Efficiency of Electronics. *Nano Energy* **2023**, *108* (August 2022), 108177. https://doi.org/10.1016/j.nanoen.2023.108177.

(8) Lin, K.; Chen, S.; Zeng, Y.; Ho, T. C.; Zhu, Y.; Wang, X.; Liu, F.; Huang, B.; Chao, C. Y.-H.; Wang, Z.; Tso, C. Y. Hierarchically Structured Passive Radiative Cooling Ceramic with High Solar Reflectivity. *Science (80-. ).* **2023**, *382* (6671), 691–697. https://doi.org/10.1126/science.adi4725.

(9) Li, T.; Zhai, Y.; He, S.; Gan, W.; Wei, Z.; Heidarinejad, M.; Dalgo, D.; Mi, R.; Zhao, X.; Song, J.; Dai, J.; Chen, C.; Aili, A.; Vellore, A.; Martini, A.; Yang, R.; Srebric, J.; Yin, X.; Hu, L. A Radiative Cooling Structural Material. *Science (80-. ).* **2019**, *364* (6442), 760–763. https://doi.org/10.1126/science.aau9101.

(10) Ghosh, P.; Wei, X.; Liu, H.; Zhang, Z.; Zhu, L. Simultaneous Subambient Daytime Radiative Cooling and Photovoltaic Power Generation from the Same Area. *Cell Reports Phys. Sci.* **2024**, *5* (3), 101876. https://doi.org/10.1016/j.xcrp.2024.101876.

(11) Heo, S.; Kim, D. H.; Song, Y. M.; Lee, G. J. Determining the Effectiveness of Radiative Cooler‐Integrated Solar Cells. *Adv. Energy Mater.* **2022**, *12* (10), 1–11. https://doi.org/10.1002/aenm.202103258.

(12) Wang, Z.; Kortge, D.; Zhu, J.; Zhou, Z.; Torsina, H.; Lee, C.; Bermel, P. Lightweight, Passive Radiative Cooling to Enhance Concentrating Photovoltaics. *Joule* **2020**, *4* (12), 2702–2717. https://doi.org/10.1016/j.joule.2020.10.004.

(13) Perrakis, G.; Tasolamprou, A. C.; Kenanakis, G.; Economou, E. N.; Tzortzakis, S.; Kafesaki, M. Passive Radiative Cooling and Other Photonic Approaches for the Temperature Control of Photovoltaics: A Comparative Study for Crystalline Silicon-Based Architectures. *Opt. Express* **2020**, *28* (13), 18548. https://doi.org/10.1364/OE.388208.

(14) Gao, K.; Shen, H.; Liu, Y.; Zhao, Q.; Li, Y.; Liu, J. Random Inverted Pyramid Textured Polydimethylsiloxane Radiative Cooling Emitter for the Heat Dissipation of Silicon Solar Cells. *Sol. Energy* **2022**, *236* (March), 703–711. https://doi.org/10.1016/j.solener.2022.03.040.

(15) Li, W.; Shi, Y.; Chen, K.; Zhu, L.; Fan, S. A Comprehensive Photonic Approach for Solar Cell Cooling. *ACS Photonics* **2017**, *4* (4), 774–782. https://doi.org/10.1021/acsphotonics.7b00089.

(16) Leroy, A.; Bhatia, B.; Kelsall, C. C.; Castillejo-Cuberos, A.; Di Capua H, M.; Zhao, L.; Zhang, L.; Guzman, A. M.; Wang, E. N. High-Performance Subambient Radiative Cooling Enabled by Optically Selective and Thermally Insulating Polyethylene Aerogel. *Sci. Adv.* **2019**, *5* (10), eaat9480. https://doi.org/10.1126/sciadv.aat9480.

(17) He, J.; Li, N.; Wang, S.; Li, S.; Wang, C.; Yu, L.; Murto, P.; Xu, X. Hygroscopic Photothermal Beads from Marine Polysaccharides: Demonstration of Efficient Atmospheric Water Production, Indoor Humidity Control and Photovoltaic Panel Cooling. *J. Mater. Chem. A* **2022**, *10* (15), 8556–8567. https://doi.org/10.1039/D2TA00594H.

(18) Li, R.; Shi, Y.; Wu, M.; Hong, S.; Wang, P. Photovoltaic Panel Cooling by Atmospheric Water Sorption–Evaporation Cycle. *Nat. Sustain.* **2020**, *3* (8), 636–643. https://doi.org/10.1038/s41893-020-0535-4.

(19) Huang, G.; Xu, J.; Markides, C. N. High-Efficiency Bio-Inspired Hybrid Multi-Generation Photovoltaic Leaf. *Nat. Commun.* **2023**, *14* (1), 3344. https://doi.org/10.1038/s41467-023-38984-7.

(20) Alezi, D.; Li, R.; Alsadun, N.; Malik, A.; Shekhah, O.; Wang, P.; Eddaoudi, M. Metal–Organic Framework-Based Atmospheric Water Harvesting for Enhanced Photovoltaic Efficiency and Sustainability. *Mater. Adv.* **2024**. https://doi.org/10.1039/D3MA00960B.

(21) Mao, Z.; Yao, Y.; Shen, J.; Liu, J.; Chen, Y.; Zhou, B.; Chen, Y.; Wang, Q.; Lu, J. Passive Interfacial Cooling-Induced Sustainable Electricity–Water Cogeneration. *Nat. Water* **2024**, *2* (1), 93–100. https://doi.org/10.1038/s44221-023-00190-6.

(22) Cheng, P.; Tang, Z.; Chen, X.; Xu, J.; Liu, P.; Zhang, X.; Wang, G. Advanced Phase Change Hydrogel Integrating Metal-Organic Framework for Self-Powered Thermal Management. *Nano Energy* **2023**, *105* (November 2022), 108009. https://doi.org/10.1016/j.nanoen.2022.108009.

(23) Xu, N.; Zhu, P.; Sheng, Y.; Zhou, L.; Li, X.; Tan, H.; Zhu, S.; Zhu, J. Synergistic Tandem Solar Electricity-Water Generators. *Joule* **2020**, *4* (2), 347–358. https://doi.org/10.1016/j.joule.2019.12.010.

(24) Alberghini, M.; Morciano, M.; Fasano, M.; Bertiglia, F.; Fernicola, V.; Asinari, P.; Chiavazzo, E. Multistage and Passive Cooling Process Driven by Salinity Difference. *Sci. Adv.* **2020**, *6* (11). https://doi.org/10.1126/sciadv.aax5015.

(25) Feng, C.; Yang, P.; Liu, H.; Mao, M.; Liu, Y.; Xue, T.; Fu, J.; Cheng, T.; Hu, X.; Fan, H. J.; Liu, K. Bilayer Porous Polymer for Efficient Passive Building Cooling. *Nano Energy* **2021**, *85* (January), 105971. https://doi.org/10.1016/j.nanoen.2021.105971.

(26) Kim, S.; Park, J. H.; Lee, J. W.; Kim, Y.; Kang, Y. T. Self-Recovering Passive Cooling Utilizing Endothermic Reaction of NH4NO3/H2O Driven by Water Sorption for Photovoltaic Cell. *Nat. Commun.* **2023**, *14* (1), 2374. https://doi.org/10.1038/s41467-023-38081-9.

(27) Liu, Y.; Liu, Z.; Wang, Z.; Sun, W.; Kong, F. Photovoltaic Passive Cooling via Water Vapor Sorption-Evaporation by Hydrogel. *Appl. Therm. Eng.* **2024**, *240* (September 2023), 122185. https://doi.org/10.1016/j.applthermaleng.2023.122185.
